# Machine learning for comprehensive forecasting of Alzheimer’s Disease progression

**DOI:** 10.1038/s41598-019-49656-2

**Published:** 2019-09-20

**Authors:** Charles K. Fisher, Aaron M. Smith, Jonathan R. Walsh, Coalition Against Major Diseases, Adam J. Simon, Chris Edgar, Clifford R. Jack, David Holtzman, David Russell, Derek Hill, Donald Grosset, Fred Wood, Hugo Vanderstichele, John Morris, Kaj Blennow, Ken Marek, Leslie M Shaw, Marilyn Albert, Michael Weiner, Nick Fox, Paul Aisen, Patricia E. Cole, Ronald Petersen, Todd Sherer, Wayne Kubick, Abbott, Alliance for Aging Research, Alzheimer’s Association, Alzheimer’s Foundation of America, AstraZeneca Pharmaceuticals LP, Bristol-Myers Squibb Company, Critical Path Institute, CHDI Foundation, Inc., Eli Lilly and Company, F. Hoffmann-La Roche Ltd, Forest Research Institute, Genentech, Inc., GlaxoSmithKline, Johnson & Johnson, National Health Council, Novartis Pharmaceuticals Corporation, Parkinson’s Action Network, Parkinson’s Disease Foundation, Pfizer, Inc., sanofi-aventis. Collaborating Organizations: Clinical Data Interchange Standards Consortium (CDISC), Ephibian, Metrum Institute.

**Affiliations:** 1 Unlearn.AI, Inc., 450 Geary St, San Francisco, CA, 94102 San Francisco, USA; 2 AJ Simon Enterprises LLC, Yardley, USA; 3 United Biosource Corporation, Blue Bell, USA; 4 Mayo Foundation for Medical Education and Research, Rochester, USA; 5 Washington University, St. Louis, USA; 6 Institute for Neurodegenerative Disorders, New Haven, USA; 7 University College London, London, UK; 8 Institute of Neurological Sciences and University of Glasgow, Glasgow, UK; 9 Octagon Research, Berwyn, PA USA; 10 Innogenetics, Ghent, Belgium; 11 Washington University, St. Louis, USA; 12 University of Goteborg, Goteborg, Sweden; 13 Institute of Neurodegenerative Disorders, New Haven, USA; 14 Department of Pathology and Laboratory Medicine, University of Pennsylvania Medical Center, Philadelphia, USA; 15 Johns Hopkins School of Medicine, Baltimore, USA; 16 University of California San Francisco, San Francisco, USA; 17 Alzheimer’s Disease Cooperative Study, La Jolla, USA; 18 ImagePace, Cincinnati, USA; 19 Mayo Clinic College of Medicine, Mayo Alzheimer’s Disease Research Center, Rochester, USA; 20 The Michael J Fox Foundation for Parkinson’s Research, New York, USA; 21 Phase Forward, Waltham, USA

**Keywords:** Machine learning, Predictive medicine

## Abstract

Most approaches to machine learning from electronic health data can only predict a single endpoint. The ability to simultaneously simulate dozens of patient characteristics is a crucial step towards personalized medicine for Alzheimer’s Disease. Here, we use an unsupervised machine learning model called a Conditional Restricted Boltzmann Machine (CRBM) to simulate detailed patient trajectories. We use data comprising 18-month trajectories of 44 clinical variables from 1909 patients with Mild Cognitive Impairment or Alzheimer’s Disease to train a model for personalized forecasting of disease progression. We simulate synthetic patient data including the evolution of each sub-component of cognitive exams, laboratory tests, and their associations with baseline clinical characteristics. Synthetic patient data generated by the CRBM accurately reflect the means, standard deviations, and correlations of each variable over time to the extent that synthetic data cannot be distinguished from actual data by a logistic regression. Moreover, our unsupervised model predicts changes in total ADAS-Cog scores with the same accuracy as specifically trained supervised models, additionally capturing the correlation structure in the components of ADAS-Cog, and identifies sub-components associated with word recall as predictive of progression.

## Introduction

Two patients with the same disease may present with different symptoms, progress at different rates, and respond differently to the same therapy. Understanding how to predict and manage differences between patients is the primary goal of precision medicine^1^. Computational models of disease progression developed using machine learning approaches provide an attractive tool to combat such patient heterogeneity. One day these computational models may be used to guide clinical decisions; however, current applications are limited both by the availability of data and by the ability of algorithms to extract insights from those data.

Most applications of machine learning to electronic health data have used techniques from supervised learning to predict specific endpoints^2–7^. An alternative to developing separate supervised models to predict each characteristic is to build a single model that simultaneously predicts the evolution of many characteristics. Statistical models based on artificial neural networks provide one avenue for developing tools that can simulate patient progression in detail^8–10^.

Clinical data present a number of challenges that are not easily overcome with current approaches to machine learning^11^. For example, most clinical datasets contain multiple types of data (i.e., they are “multimodal”), have a relatively small number of samples, and many missing observations. Dealing with these issues typically requires extensive preprocessing^3^ or simply discarding variables that are too difficult to model. For example, one recent study focused on only four variables that were frequently measured across all 200,000 patients in an electronic health dataset from an intensive care unit^9^. Developing methods that can overcome these limitations is a key step towards broader applications of machine learning in precision medicine.

Precision medicine is especially important for complex disorders in which patients exhibit different patterns of disease progression and therapeutic responses. Alzheimer’s Disease (AD) and Mild Cognitive Impairment (MCI) are complex neurodegenerative diseases with multiple cognitive and behavioral symptoms^12^. The severity of these symptoms is usually assessed through exams such as the Alzheimer’s Disease Assessment Scale (ADAS)^13^ or Mini Mental State Exam (MMSE)^14^. The heterogeneity of AD and related dementias makes these diseases difficult to diagnose, manage, and treat, leading to calls for better methods to forecast and monitor disease progression and to improve the design of AD clinical trials^15^. The challenge of distinguishing between related disorders makes differential diagnosis also of interest^16^.

A variety of disease progression models have been developed for MCI and AD using clinical data^17–21^ or imaging studies^22–31^. Although previous approaches to forecasting disease progression have proven useful^32,33^, they have focused on predicting a single endpoint, such as the change in the ADAS Cognitive (ADAS-Cog) score from baseline. Given that AD is heterogeneous and multifactorial, we set out to model the progression of more than just the ADAS-Cog score. We accomplished this by simulating the progression of entire patient profiles, describing the evolution of each sub-component of the ADAS-Cog and MMSE scores, laboratory tests, and their associations with baseline clinical characteristics.

The manuscript is structured as follows. Section 2.1 describes our dataset and Section 2.2 describes our machine learning model. Section 2.3 assesses the goodness-of-fit of our machine learning model. Predictions for individual components are discussed in Section 2.4. Section 2.5 assesses the accuracy of our approach, which simulates each sub-component of the cognitive scores, at predicting changes in overall disease activity measured by the ADAS-Cog exam. Finally, Section 3 discusses implications.

## Results

### Data

Our statistical models were trained and tested on data extracted from the Coalition Against Major Diseases (CAMD) Online Data Repository for AD (CODR-AD)^34,35^. We extracted 18-month longitudinal trajectories of 1909 patients with MCI or AD covering 44 variables including the individual components of the ADAS-Cog and MMSE scores, laboratory tests, and background information. Each patient profile consisted of 44 covariates (Table 1) that were classified as binary, ordinal, categorical, or continuous. Patient trajectories described the time evolution of all 44 variables in 3-month intervals. Detailed data processing steps are described in Section 5.1 and in the Supporting Information.

**Table 1:** The model includes variables assessing cognitive function (ADAS and MMSE), as well as laboratory, clinical, and background variables.

Name	Category	Type	Temporal	Statistics	Missing%
Commands	ADAS	Ordinal	Yes	0.58 (0.84)	0.1
Comprehension				0.40 (0.72)	0.1
Construction				0.94 (0.90)	0.1
Delayed Word Recall				7.94 (2.51)	0.4
Ideational				0.54 (0.88)	0.1
Instructions				0.78 (1.18)	0.1
Naming				0.67 (0.88)	0.1
Orientation				2.48 (2.02)	0.1
Spoken Language				0.30 (0.69)	0.1
Word Finding				0.66 (0.90)	0.1
Word Recall				6.04 (1.78)	0.1
Word Recognition				6.35 (3.30)	0.1
Attention and Calculation	MMSE	Ordinal	Yes	2.88 (1.69)	16.8
Language				7.90 (0.92)	16.8
Orientation				6.56 (1.92)	16.8
Recall				0.82 (0.88)	16.8
Registration				2.90 (0.34)	16.8
Alanine aminotransferase	Laboratory	Continuous	Yes	0.32 (0.14) *μ*kat/l	18.2
Alkaline phosphatase				1.29 (0.46) *μ*kat/l	18.2
Aspartate aminotransferase				0.37 (0.10) *μ*kat/l	18.2
Cholesterol				5.5 (1.0) mmol/l	17.9
Creatine kinase				0.99 (0.62) mg/dl	0.7
Creatinine				0.95 (0.22) mg/dl	17.9
Gamma glutamyl transferase				2.3 (1.8) iu/dl	32.0
Hematocrit				0.42 (0.04) counts	14.7
Hemoglobin				14.0 (1.2) g/dl	0.8
Hemoglobin a1c				5.81 (0.73)%	48.4
Indirect bilirubin				0.51 (0.24) mg/dl	48.4
Potassium				4.34 (0.35) mmol/l	18.0
Sodium				1.41 (0.02) mmol/cl	31.8
Triglycerides				1.53 (0.83) g/l	18.1
Blood pressure (diastolic)	Clinical	Continuous	Yes	75.9 (8.3) mmHg	1.8
Blood pressure (systolic)		Continuous		135 (15) mmHg	1.8
Heart rate		Continuous		67.3 (8.2) bpm	1.8
Weight		Continuous		71 (15) kg	3.0
Dropout		Binary		none	0.1
Age at baseline	Background	Continuous	No	73.4 (8.4) years	0.9
Geographic region		Categorical		67% North America	0
Initial diagnosis (AD or MCI)		Binary		69% AD/31% MCI	0
Past cardiovascular event		Binary		37% Y/63% N	0
ApoE *ε*4 allele count		Ordinal		36% 0/48% 1/16% 2	72.4
Race		Categorical		93% White	0.2
Sex		Binary		54% F/46% M	0
Height		Continuous		165 (10) cm	1.9

The statistics column gives the mean and standard deviation of the data (combining training, validation, and test data) at baseline, along with any units. For geographic region and race, the dominant category frequency is given. The missing percentage column gives the percentage of missing data for each variable at baseline.

### Modeling with Conditional Restricted Boltzmann Machines

A statistical model is generative if it can be used to draw new samples from an inferred probability distribution. Generative modeling of clinical data involves two tasks: i) randomly generating patient profiles with the same statistical properties as real patient profiles and ii) simulating the evolution of these patient profiles through time. Each of these tasks is complicated by common properties of clinical data, namely that they are typically multimodal and have many missing observations. Moreover, patient progression is best regarded as a stochastic process and it is important to capture the inherent randomness of the underlying processes in order to make accurate forecasts.

Let **x**_*i*_(*t*) be a vector of covariates measured in patient *i* at time *t*. Creating a generative model to solve (i) involves finding a probability distribution *P*(**x**) such that we can randomly draw **x**_*i*_(*t* = 0) ~ *P*(**x**). Solving problem (ii) involves finding a conditional probability distribution *P*(**x**(*t*))|**x**(*t* − 1) so that we can iteratively draw **x**_*i*_(*t*) ~ *P*(**x**_*i*_(*t*))|**x**_*i*_(*t* − 1) to generate a patient trajectory.

Our statistical model for patient progression is a latent variable model called a Conditional Restricted Boltzmann Machine (CRBM)^36–39^. A CRBM is an undirected neural network capable of learning and sampling from the joint probability distribution of covariates across multiple times. To construct the model, the covariates were divided into two mutually exclusive subsets: *static* covariates that were determined solely from measurements at the beginning of the study ${{\bf{x}}}_{i}^{{\rm{static}}}(t=0)$, and *dynamic* covariates that changed during the study ${{\bf{x}}}_{i}^{{\rm{dynamic}}}(t)$. To train the model, we defined vectors ${{\bf{v}}}_{i}(t)=\{{{\bf{x}}}_{i}^{{\rm{dynamic}}}(t),{{\bf{x}}}_{i}^{{\rm{dynamic}}}(t-1),{{\bf{x}}}_{i}^{{\rm{static}}}(t=0)\}$ by concatenating neighboring time points with the static covariates. All neighboring time points are combined into a single dataset used to train a single statistical model that applies to all neighboring time points. Rather than directly modeling the correlations between these covariates, a CRBM models these correlations indirectly using a vector of latent variables **h**_*μ*_(*t*). These latent variables can be interpreted in much the same way as directions identified through principal components analysis.

The CRBM is a parametric statistical model for which the probability density is defined as

1 $$p({\bf{v}},{\bf{h}})={Z}^{-1}\exp (\sum _{j}{a}_{j}({v}_{j})+\sum _{\mu }{b}_{\mu }({h}_{\mu })+\sum _{j\mu }{W}_{j\mu }\frac{{v}_{j}}{{\sigma }_{j}^{2}}\frac{{h}_{\mu }}{{\varepsilon }_{\mu }^{2}}),$$

and *Z* is a normalization constant that ensures the total probability integrates to one. Here, *a*_*j*_(*v*_*j*_) and and *b*_*μ*_(*h*_*μ*_) are functions that characterize the data types of covariate *v*_*j*_ and latent variable *h*_*μ*_, respectively. The parameters *σ*_*j*_ and *ε*_*μ*_ set the scales of *v*_*j*_ and *h*_*μ*_, respectively. We used 50 normally distributed latent variables that were lower truncated at zero, which is known as a rectified linear (ReLU) activation function in the machine learning literature^40^. To deal with missing data, we divide the visible vector **v** into mutually exclusive groups **v**_*missing*_ and **v**_*observed*_ and impute the missing values by drawing from the conditional distribution *p*(**v**_*missing*_|**v**_*observed*_).

**Figure 1:**
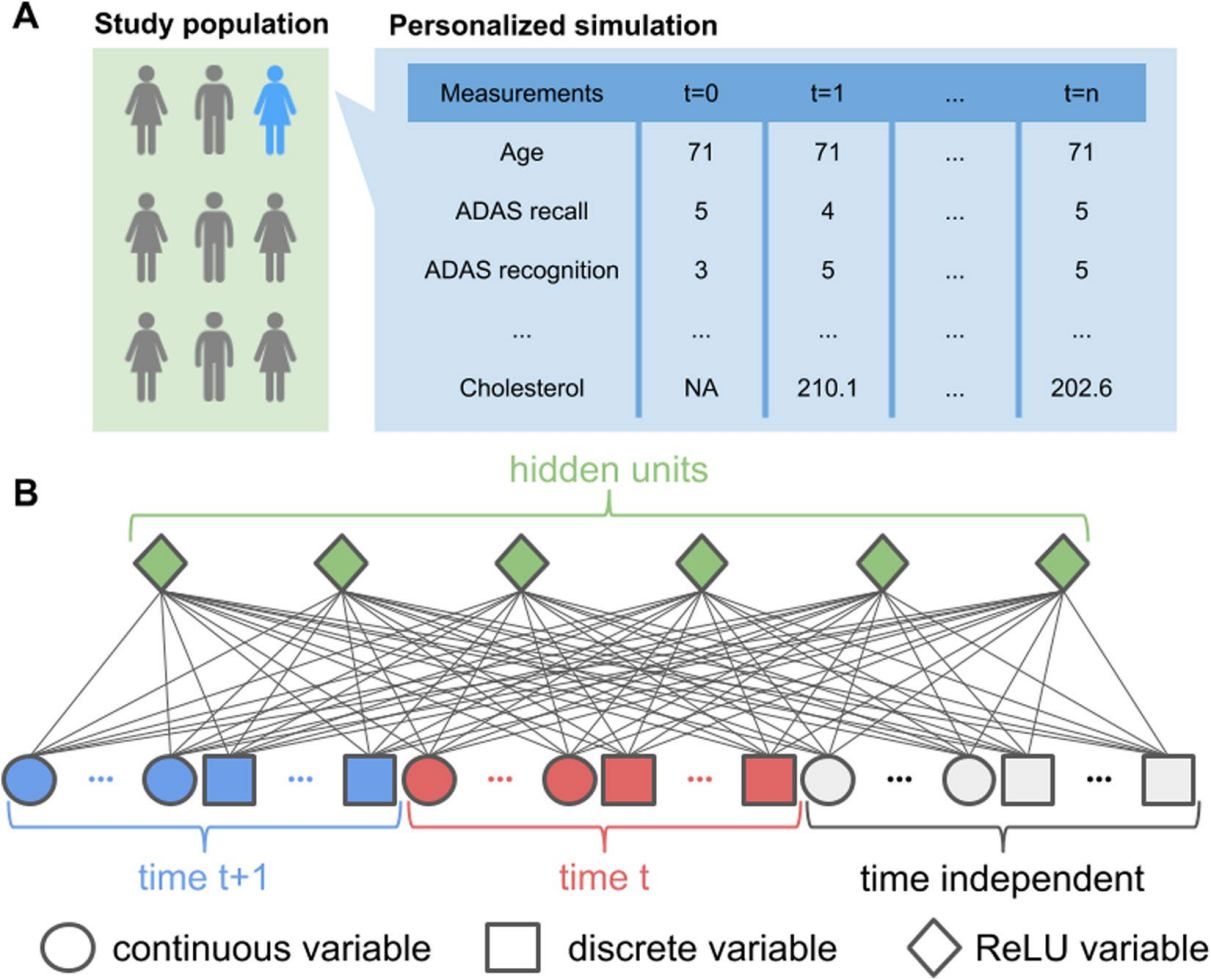
Overview of the data and model. (**A**) Study data built from the CAMD database consists of 18-month longitudinal trajectories of 1909 patients with MCI or AD. Our model uses 44 variables, including the individual components of the ADAS-Cog and MMSE scores, laboratory tests, and background information. (**B**) To capture time dependence, we model the joint distribution of the data at time *t* + 1 and the data at time *t* using a Conditional Restricted Boltzmann Machine (CRBM) with ReLU hidden units. Multimodal observations are modeled with different types of units in the visible layer and missing observations are automatically imputed.

Traditionally, CRBMs are trained to maximize the likelihood of the data under the model using stochastic maximum likelihood^41^. Recent results have shown that one can improve on maximum likelihood training of RBMs by adding an additional term to the loss function that measures how easy it is to distinguish patient profiles generated from the statistical model from real patient profiles^42^. Therefore, we used a combined maximum likelihood and adversarial training method to fit the CRBM; more details of the machine learning methods are described in the Supporting Information. An overview of our statistical model is depicted in Fig. 1.

To explore and better quantify the performance of the CRBM, we used 5-fold cross validation (CV) for the analysis. On each of 5 folds a CRBM was trained on 80% of patients (75% for training, 5% for validation), and the remaining 20% was used to test that CRBM. In the analysis, results over the 5 folds were either averaged (and the standard deviation over the folds used as an uncertainty), or aggregated (e.g., for plotting distributions over the test data). Every result shown is on out-of-sample test data.

We generated two types of synthetic patient trajectories with the trained CRBMs: (i) synthetic trajectories starting from baseline values for real patients, and (ii) entirely synthetic patients. The first type is useful for many tasks in precision medicine and clinical trial simulation, while the second type has interesting applications for maintaining the privacy of clinical data^43^. To generate trajectories of type (i), an initial population of patients was selected and then the model was used to predict their future state. To accomplish this, we started with baseline data and used the CRBM to iteratively add new time points. To generate trajectories of type (ii), entirely synthetic patients were generated by first simulating the baseline data, then iteratively adding new time points so that the patient data was entirely simulated for the full trajectory.

### Goodness-of-fit of the model

The fundamental assumption underyling our analysis is that each time-dependent variable in a patient’s clinical record is *stochastic*; it does not take on a single deterministic value, but is sampled from a distribution of values. For example, if we had the ability to repeatedly measure the cognitive function of a particular patient 12 months from a baseline measurement, we would not observe the same value every time but would instead observe a distribution of values. A CRBM describes this time-dependent probability distribution associated with a patient’s characteristics. If we could actually perform this thought experiment, then we could compare the distribution of values observed for a particular patient at each time point to the distribution predicted by the model in order to assess how well the model fits the data. In practice, of course, we are only able to observe one draw from each patient’s distribution. Therefore, we use a variety of metrics to assess if the time-dependent means, standard deviations, correlations, etc, determined from the model are consistent with those observed in the test dataset.

To make these comparisons, we used the first type of synthetic patient trajectories described in the previous section. Starting with the baseline values for actual patients, we simulated the trajectory of these patients beyond baseline. We repeated this many times to measure the distribution of each covariate at each timepoint for each patient. All of the actual patients were taken from the test dataset associated with the appropriate CV fold.

**Figure 2:**
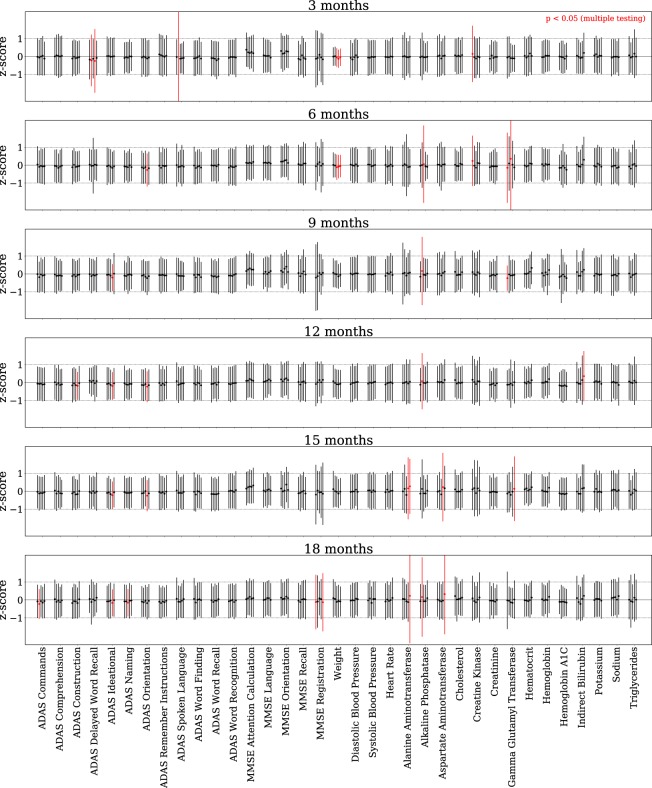
The model accurately simulates individual patient trajectories. The mean and variance over all patients of the per-patient z-score distribution is shown for every time-dependent variable (except dropout) at every time point beyond baseline. Results are shown for all CV models. For each patient, the z-score is calculated from 100 simulations of that patient conditioned on the baseline data (the synthetic subjects are of type (i) described above). The first (mean) and second (standard deviation) moments of the distribution of z-scores over all patients for each variable and time point is computed and displayed with a point (mean) and error bar (standard deviation). Under the assumption that a variable is normal, this z-score distribution should be a standard normal, with mean 0 and standard deviation 1. For each variable at each time point, we use the Kolmogorov-Smirnov test to evaluate whether the mean and standard deviation of the z-score distribution are significantly different from a standard normal. Any significantly different (*p* < 0.05) cases remaining so after a Bonferroni correction are labeled in red.

First, we focus on assessing the time-dependent means and standard deviations computed from a CRBM. For a particular patient *i*, the observed value of variable *j* at time *t* is *x*_*ij*_(*t*). The conditional mean and variance computed from the CRBM are denoted ${\rm{E}}[{x}_{ij}(t)|{{\bf{x}}}_{i}(0)]$ and ${\rm{Var}}[{x}_{ij}(t)|{{\bf{x}}}_{i}(0)]$, respectively. Because we only have a single observation for any given patient, we had to aggregate data across patients in order to perform any statistical comparisons. To do so, we computed a z-score

2 $${z}_{ij}(t)=\frac{{x}_{ij}(t)-{\bf{E}}[{x}_{ij}(t)|{{\bf{x}}}_{i}(0)]}{\sqrt{{\rm{Var}}[{x}_{ij}(t)|{{\bf{x}}}_{i}(0)]}}$$

by subtracting the predicted mean and dividing by the predicted standard deviation for each observed data point. If the predicted means and standard deviations are consistent with the data then *z*_*ij*_(*t*) will have zero mean and unit standard deviation when viewed across all of the patients (i.e., taking the average with respect to the patient index *i*). The computed means and standard deviations of the z-scores for each time-dependent variable are shown in Fig. 2, where they are compared to the ideal values of zero and one, respectively.

We made a simplifying assumption to enable us to compute p-values for each of these comparisons. If the actual conditional distribution of *x*_*ij*_(*t*) were normal with mean ${\rm{E}}[{x}_{ij}(t)|{{\bf{x}}}_{i}(0)]$ and variance ${\rm{Var}}[{x}_{ij}(t)|{{\bf{x}}}_{i}(0)]$, then ${z}_{ij}(t) \sim {\mathscr{N}}(0,1)$ would be drawn from a standard normal distribution. As above, we aggregate *z*_*ij*_(*t*) across patients in order to gain enough observations in order to perform a statistical test to determine if the moments computed from the CRBM for variable *j* at time *t* are consistent with those observed in the test set. We computed the Kolmogorov-Smirnov test statistic for the mean *μ* and standard deviation *σ*, ${D}_{KS}(\mu ,\sigma )={{\rm{\sup }}}_{x}|\Phi (x;\mu ,\sigma )-\Phi (x;0,1)|$, and computed a p-value from the Kolmogorov distribution using the test statistic $\sqrt{n}{D}_{KS}$. Differences that were significant at *p* < 0.05 and survive a Bonferroni multiple-testing correction^44^ are marked in red. A non-significant p-value for a variable implies that the per-patient distribution of that variable obtained from the CRBM has a mean and standard deviation that are consistent with the data. The fact most variables do not show statistically significant differences is a clear indicator of the accuracy of the CRBM. Additional comparisons of univariate distributions are provided in Supplementary Figs S1–S4, and Supplementary Fig. S5 shows a detailed comparison between the data and CRBM for the first and second moment statistics of each variable and each time point.

**Figure 3:**
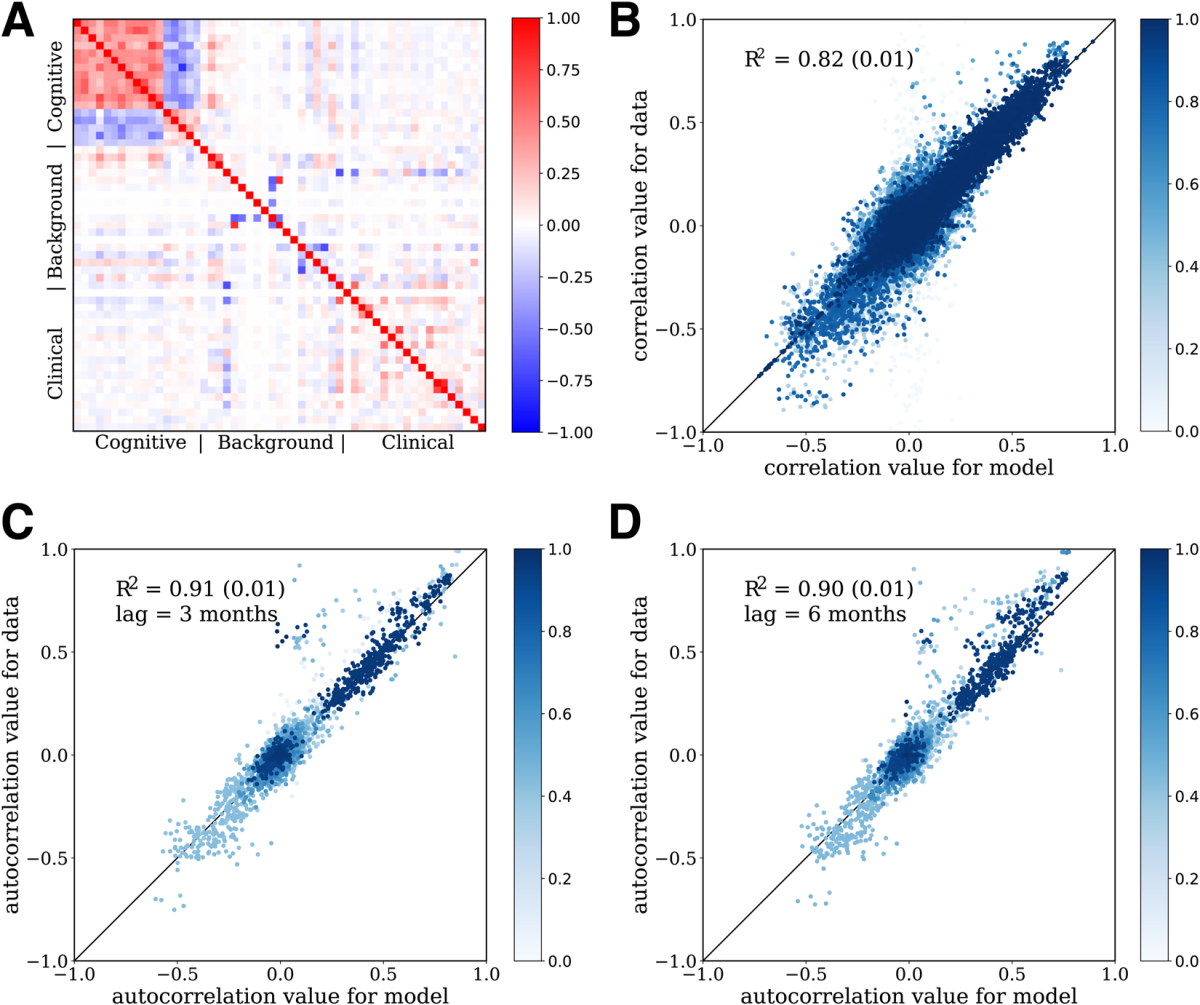
The CRBM models correlations well. (**A**) Correlations between variables as predicted by the model (below the diagonal) and calculated from the data (above the diagonal). Components of the cognitive scores are strongly correlated with each other, but not with other clinical data. (**B**) Scatterplot of observed vs predicted correlations for each time point, over all times. (**C**) Scatterplot of observed vs predicted autocorrelations with time lag of 3 months. (**D**) Scatterplot of observed vs predicted autocorrelations with time lag of 6 months. The color gradient in (**B**–**D**) represents the fraction of observations for which the variables used to compute the correlation were present; lighter colors mean more of the data was missing. In all cases, synthetic patients conditioned on baseline data from actual patients is used (synthetic patients of type (i) above). In (**A**), the correlation coefficients shown are averaged over the 5 CV models. In (**B**–**D**), the correlation coefficients for each of the 5 CV folds are shown, and the *R*^2^ values shown are the mean and standard deviation over the 5 CV folds, computed from a least squares fit weighted by the fraction of data present when computing the correlations. In all cases the correlations for data are only computed on samples for which the relevant variables are both present (i.e., missing data is ignored).

Next, we move beyond univariate statistics to assess if the CRBM correctly captures the correlations between the variables. Figure 3A shows that many pairs of variables are indeed correlated, so modeling these correlations is non-trivial. These equal-time correlations suggest that the variables can be grouped into three categories: cognitive scores, laboratory and clinical tests, and background information. There are strong correlations between variables belonging to the same category but only weak inter-category correlations. Figure 3B shows a comparison of the pairwise correlations computed from the model with those computed from the test data for all CV folds, with an *R*^2^ = 0.82 ± 0.01.

In addition to measuring the correlations between pairs of variables at the same time, one can measure correlations between pairs of variables at different times to get an idea for how the variables change over time. Comparisons between time-lagged correlations computed from the model and from the test data are shown in Fig. 3C for a 3 month time lag (*R*^2^ = 0.91 ± 0.01) and Fig. 3D for a 6 month time lag (*R*^2^ = 0.90 ± 0.01). The good agreement between the data and model for the 6 month time lag correlations is an important check because the CRBM only includes parameters to account for the 3 month autocorrelations.

It is important to note that missing data can affect the ability to estimate correlations between variables. Imputation of missing data was not performed for the statistics calculated on the data; instead, only the samples in which both variables were present were used to compute a correlation. The fraction of time a pair of variables was present is represented in Fig. 3B–D with a blue color gradient. In addition, the *R*^2^ was computed using a weighted regression in which the weights on each correlation were determined by the fraction of data present in the computation.

**Figure 4:**
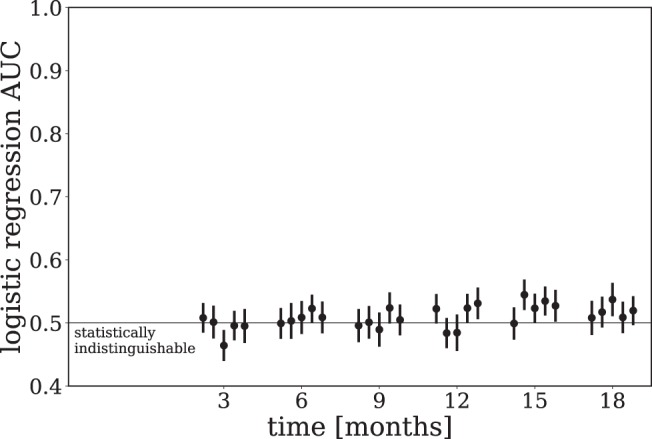
Synthetic data is challenging to distinguish from real data. The AUC of a logistic regression model trained at each time point to distinguish between real and synthetic data is shown. At each time point a dataset is formed comprised of real and synthetic patient data in which the baseline data for each group is the same. A logistic regression model is trained to separate these two groups, and the performance using the AUC metric is estimated with 5-fold cross validation. This procedure is repeated many (100) times and the average AUC is shown along with the standard deviation. Finally, this entire method is repeated for each CV fold (which are all shown). To handle missing data mean imputation is used, with the corresponding entries in the synthetic data also assigned the same values. The performance of the logistic regression at each time point is consistent with statistically indistinguishable real and synthetic data.

As a final test of goodness-of-fit, we evaluated the ability of logistic regression to differentiate actual and synthetic patient data at each time point beyond baseline. At each time point we compared actual and synthetic patient data in which each synthetic patient was conditioned on the corresponding actual patient’s baseline data. A logistic regression model was trained to differentiate these two groups of patients, and the performance was estimated using the Area Under the receiver operating characteristic Curve (AUC) metric computed using 5-fold cross validation. Note that this type of analysis is commonly employed to assess differences between populations using propensity score matching^45^. The AUC was averaged over 100 simulations from the CRBM, with the mean and standard deviation for each CV fold shown in Fig. 4. For all points, the AUC of the logistic regression model is consistent with a score of 0.5, meaning the logistic regression model cannot reliably distinguish between actual and synthetic patient data at any timepoint.

Figures 2, 3 and 4 quantitatively assess the accuracy of the CRBM, directly comparing actual and synthetic patient data. These figures demonstrate the model is accurately predicting the first and second moments and correlations of the distribution of actual patient data, even at the per-patient level. The equal-time and lagged autocorrelations between variables as well as the mean and standard deviation of each variable at each time point are all well modeled. Additionally, a standard linear classifier is unable to distinguish actual and synthetic patient data at each time point beyond baseline. We now turn our attention to comparing the performance of the CRBM to other models and examining the ways the model may be applied to patient data.

### Simulating conditional patient trajectories

Predictions for any unobserved characteristics of a patient can be computed from our model by generating samples from the model distribution conditioned on the values of all observed variables. Sampling from the conditional distributions can be used to fill-in any missing observations (i.e., imputation) or to forecast a patient’s future state. The ability to sample from any conditional distribution is one advantage a modeling framework based on CRBMs has over alternative generative models based on directed neural networks.

A CRBM is designed to capture the underlying time-dependent probability distribution of values under the assumption that disease progression is a stochastic process. To distill this distribution into a single ‘predicted’ value for variable *j* in patient *i* at time *t*, we computed the conditional expectation ${\rm{E}}[{x}_{ij}(t)|{{\bf{x}}}_{i}(t=0)]$, which is the minimum mean squared error predictor for *x*_*ij*_(*t*) under the model.

**Figure 5:**
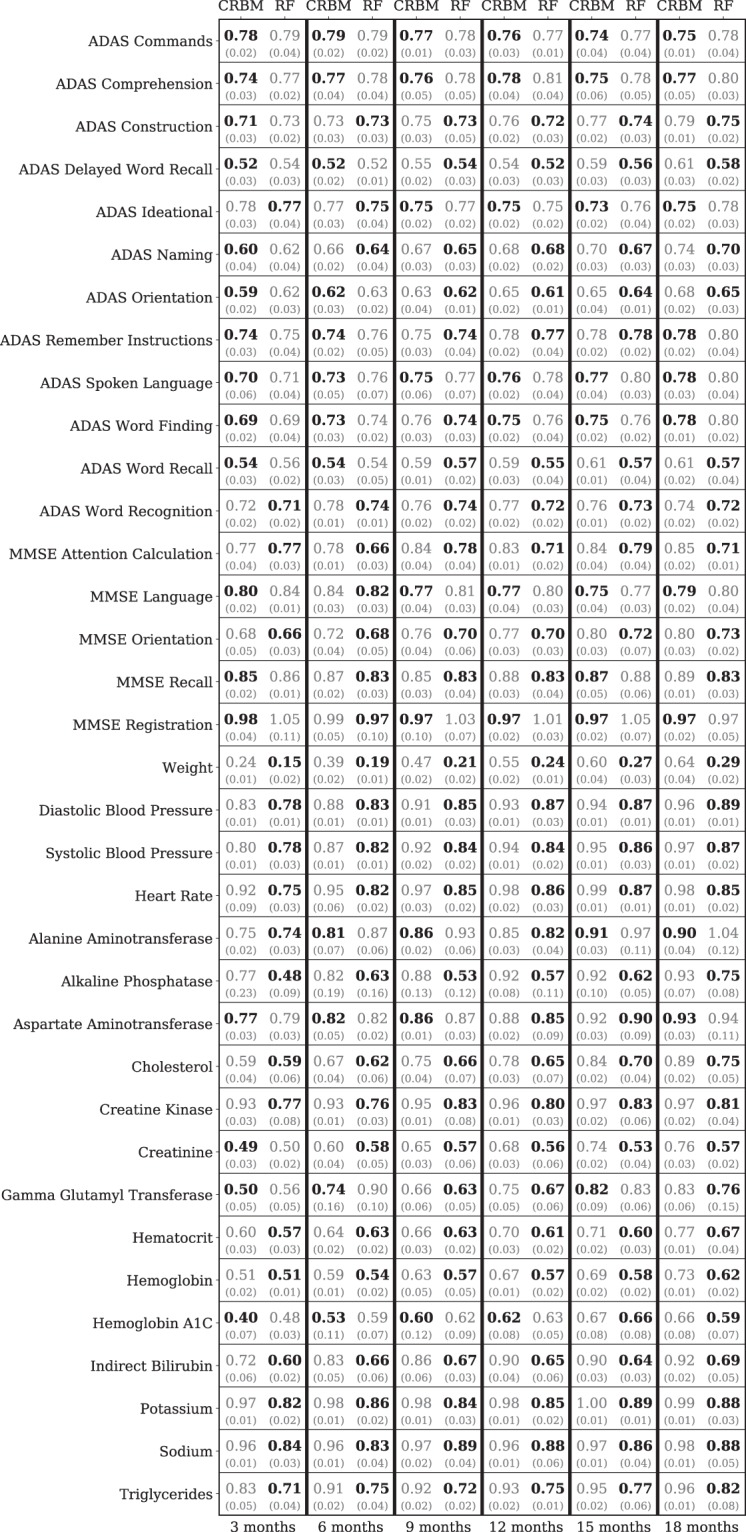
The model accurately forecasts across variables. Relative errors of the model (CRBM) and a random forest (RF) specifically trained to predict the value of a single variable at a single time point. The root mean square (RMS) errors are scaled by the standard deviation of the data to be predicted. The mean (top number) and standard deviation (bottom number, in parentheses) of these scaled errors over the 5 CV folds are shown. Predictions are shown for every time-dependent variable except dropout. At each time point and for each variable, the better of the random forest and CRBM predictions is shown in bold.

For comparison, we trained a series of Random Forest (RF) models that use the baseline data to predict each of the 35 time-dependent variables for all 6 time points. Note that there is a separate RF model for each variable at each time point – a total of 210 different RF models. We also trained an ensemble of 6 multivariate RFs – each one predicted all 35 covariates for a given time point – but were unable to get reasonable accuracies (see Supporting Information). For each RF model, mean imputation was used to replace missing data; when the dependent variable to be predicted was missing for a sample, that sample was excluded for both RF and CRBM models. The RMS error of the random forest prediction sets a benchmark for a predictive model that is specially trained for an individual problem. By contrast, a single CRBM model is used to predict all variables, and all time points. Figure 5 presents a detailed comparison between the single CRBM and the ensemble of 210 RF models. The accuracy of the CRBM is close to the specialized RF model for each variable and time point, with the CRBM performing best relative to the RF on the components of ADAS-Cog and more poorly on the non-cognitive variables.

**Figure 6:**
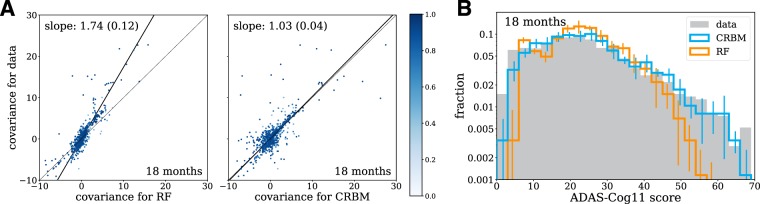
The model accurately captures statistics of variables for which supervised methods do not. (**A**) The covariance values between the model (CRBM) and the random forests (RF) compared to the data. Theil-Sen estimators for the slope of the covariance values in the data relative to the CRBM and RF are shown. Large covariance values are off-scale and not shown on the figure. (**B**) The distribution of ADAS-Cog scores for the data, the model (CRBM), and the random forests (RF). The random forest models shown are the same models trained to predict single variables at single time points, shown in Fig. 5. The CRBM is conditioned on the baseline data and simulates the 18-month data, while the RF models predict the 18-month data from the baseline data. In both cases the CRBM accurately captures the statistics of the data, while the RF under-predicts the covariance values and the extent of the ADAS-Cog distribution. In both (**A**) and (**B**), the data from all 5 CV folds are shown together, and in (**A**) the Theil-Sen slope is computed on this combined dataset. The errors in the Theil-Sen slope and the error bars in (**B**) are standard deviations across the 5 CV folds.

As with most supervised models, a decision tree in a RF is trained to minimize the mean squared error. Therefore, a RF learns a function ${f}_{jt}({\bf{x}}(0))\approx {\rm{E}}[{x}_{ij}(t)|{{\bf{x}}}_{i}(t=0)]$. With that in mind, it is not surprising that the performance of the mean computed from the CRBM and the prediction from the ensemble of RFs have similar mean squared errors. This also means that, unlike a CRBM, the RF cannot generate realistic trajectories that capture the correlations between the covariates. Figure 6A shows that the RF ensemble under-predicts covariance values, as evidenced by the slope of the outlier-robust Theil-Sen regression between the data and the RF ensemble. By comparison, the CRBM is in much better agreement with the covariances computed from the data.

The difference between the higher-order statistics computed from the RF ensemble and the CRBM can be understood in terms of the law of total variance. If **x**(*t*) are the covariates at time *t*, then the law of total variance divides the covariance values into two contributions conditioned upon baseline covariates:

3 $${\rm{Cov}}[{\bf{x}}(t)]={\rm{Cov}}[{\rm{E}}[{\bf{x}}(t)|{\bf{x}}(t=0)]]+{\rm{E}}[{\rm{Cov}}[{\bf{x}}(t)|{\bf{x}}(t=0)]]$$

Samples drawn from the CRBM reflect both terms, but deterministic predictions from the RF ensemble neglect contributions to the total covariance arising from the second term. Figure 6B illustrates this for distribution of the ADAS-Cog11 score. Treating the predictions of the RF ensemble as trajectories would lead one to underestimate the variance of the distribution, particularly in the right tail. By contrast, the distribution computed from the CRBM fits the observed distribution quite well. More details on the comparison between RFs and the CRBM are provided in the Supporting Information.

In summary, stochastic simulations of disease progression have two main advantages compared to supervised machine learning models that aim to predict a single, predefined endpoint. The first is that the simultaneous modeling of entire patient profiles captures correlations between the covariates. This allows for the quantitative exploration of alternative endpoints and different patient subgroups. The second is that stochastic simulations provide in-depth estimates of risk for individual patients that can be aggregated to estimate risks in larger patient populations. Moreover, our model provides accurate estimates of variance in addition to forecasts for expected progression of individual patients (Figs S1 and S10).

### Forecasting and interpreting disease progression

**Figure 7:**
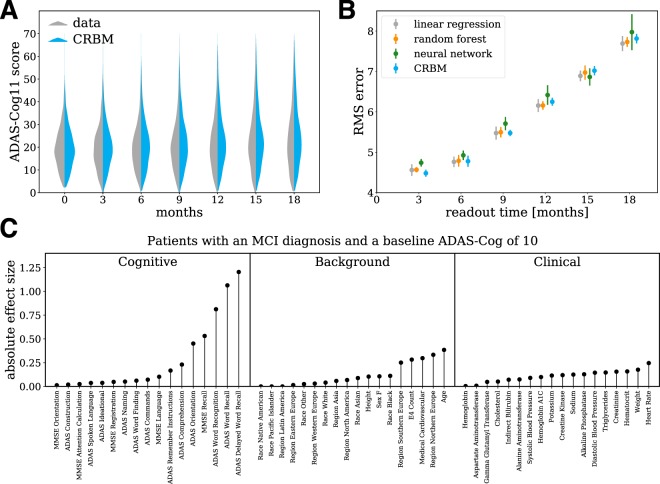
The model accurately forecasts progression and allows for interpretation. (**A**) Violin plot of the ADAS-Cog score over time computed from the data and the model. The data from all 5 CV folds are shown together. (**B**) Out-of-sample predictive accuracy for the change in ADAS-Cog score from baseline (i.e., *t* = 0) for different study durations. Separate neural network, random forest, and linear regression models were trained to predict the change in ADAS-Cog score from baseline for each study duration. The points (errors) are the means (standard deviations) over the 5 CV folds. (**C**) We created a simulated patient population with MCI and an initial ADAS-Cog score of 10, and simulated the evolution of each synthetic patient for 18 months. The 5% of synthetic patients with the largest ADAS-Cog score increase were designated “fast progressors” and the bottom 5% of patients with the smallest ADAS-Cog score increase were designated “slow progressors”. Differences between the fast and slow progressors (the “absolute effect size”) were quantified using the absolute value of Cohen’s *d*-statistic, which measures the mean difference divided by a pooled standard deviation^47^. The average effect size over the 5 CV folds is shown for each variable.

In this last section, we focus on disease progression as assessed by the overall ADAS-Cog11 score rather than the individual components. Our model is trained to simulate the evolution of the individual components of the cognitive exams, laboratory tests, and clinical data. As a result, it is also possible to simulate the evolution of any combination of these variables, such as the 11-component ADAS-Cog score that is commonly used as a measure of overall disease activity. Note that the ADAS delayed word recall component, which is present in the dataset, is not part of the 11-component ADAS-Cog score but can be used as an additional probe of disease severity, especially for MCI^46^. Figure 7A shows a violin plot describing the evolution of the ADAS-Cog score distribution within the population. The data and model show the same trend – an increase in the mean ADAS-Cog score with time along with a widening right tail of the distribution. This implies that much of the trend of increasing ADAS-Cog scores in the population is driven by a subset of patients.

As in the previous section, the CRBM can be used to compute the mean ADAS-Cog11 score for a patient conditioned on the baseline measurements of each variable. In Fig. 7B, we have compared the accuracy of the CRBM predictions for the change in ADAS-Cog11 score from baseline to each possible endpoint in 3-month steps through 18 months to a variety of supervised models (a linear regression, a random forest, and a deep neural network). The figure shows the root-mean-square error (RMS error) of each model’s prediction for the 18-month change ADAS-Cog11 score. The figure shows the mean value and standard deviation over all 5 CV folds. Each of the supervised models was trained to predict a specific endpoint (e.g., the change in ADAS-Cog score after 6 months). The CRBM has equivalent performance to these models over the entire range. That is, despite only being trained on data on the individual components with a 3-month time lag, the mean ADAS-Cog11 score computed from the CRBM is as accurate as supervised models trained only for this task. More details on the comparison are provided in the Supporting Information.

To gain more insight into the origin of fast and slow progressing patients, we simulated 18-month patient trajectories conditioned on a baseline ADAS-Cog11 score of 10 and an initial diagnosis of MCI. This initial ADAS-Cog11 score was chosen because it is representative of a typical patient with MCI. The 5% of synthetic patients with the largest ADAS-Cog11 score increase were designated “fast progressors” and the bottom 5% of synthetic patients with the smallest ADAS-Cog11 score increase were designated “slow progressors”. Differences in baseline characteristics between the fast and slow progressors (the “absolute effect size”) were quantified using the absolute value of Cohen’s *d*-statistic^47^, as shown in Fig. 7C. The majority of baseline variables are not associated with disease progression; however, there are strong associations with cognitive tests based on recall (i.e., MMSE recall, ADAS word recall, and ADAS delayed word recall) and word recognition. That is, patients with poor performance on the ADAS delayed word recall test tend to progress more rapidly – even after controlling for the total ADAS-Cog11 score. Variables associated with progression in patients who already have AD are described in the Supporting Information.

## Discussion

The ability to simulate the stochastic disease progression of individual patients in high resolution could have a transformative impact on patient care by enabling personalized data-driven medicine. Each patient with a given diagnosis has unique risks and a unique response to therapy. Due to this heterogeneity, predictive models cannot currently make individual-level forecasts with a high degree of confidence. Therefore, it is critical that data-driven approaches to personalized medicine and clinical decision support provide estimates of variance in addition to expected outcomes.

Previous efforts for modeling disease progression in AD have focused on predicting changes in predefined outcomes such as the ADAS-Cog11 score or the probability of conversion from MCI to AD^17–25,27–31^. Here, we have demonstrated that an approach based on unsupervised machine learning can create stochastic simulations of entire patient trajectories that achieve the same level of performance on individual prediction tasks as specific models while also accurately capturing correlations between variables. Machine learning-based generative models provide much more information than specific models, thereby enabling a simultaneous and detailed assessment of different risks.

Our approach to modeling patient trajectories in AD overcomes many of the limitations of previous applications of machine learning to clinical data^3,8,9,11^. CRBMs can directly integrate multimodal data with both continuous and discrete variables, and time-dependent and static variables, within a single model. In addition, bidirectional models like CRBMs can easily handle missing observations in the training set by performing automated imputation during training. Combined, these factors dramatically reduce the amount of data preprocessing steps needed to train a generative model to produce synthetic clinical data. We found that a single time-lagged connection was sufficient for explaining temporal correlations in AD; additional connections may be required for diseases with more complex temporal evolution.

The utility of cognitive scores as a measure of disease activity for patients with AD has been called into question numerous times^48^. Here, we found that the components of the ADAS-Cog and MMSE scores were only weakly correlated with other clinical variables. One possible explanation is that the observed stochasticity may simply reflect heterogeneity in performance on the cognitive exam that cannot be predicted from any baseline measurements. However, we did find that some of the individual components of the baseline cognitive scores are predictive of progression. Specifically, patients with poor performance on word recall tests tend to progress more rapidly than other patients, even after controlling for the ADAS-Cog11 score.

There are a number of improvements to our dataset and methodology that are important steps for future research. Here, we limited ourselves to modeling 44 variables that are commonly measured in AD clinical trials. We excluded some interesting covariates such as Leukocyte populations because they were not measured in the majority of patients in our dataset constructed from the CAMD database. We also lack data from neuroimaging studies and tests for levels of amyloid-*β*. Incorporating additional data into our model development will be a crucial next step, especially as surrogate biomarkers become a standard part of clinical trials.

## Conclusions

This work provides a proof-of-concept that patient-level simulations are technologically feasible with the right tools and data. We have shown that generative models capable of sampling conditional probability distributions over a diverse array of clinical variables can accurately model the progression of Alzheimer’s Disease. These models have been broadly validated, from the ability to capture statistics of distributions of clinical variables to their ability to predict progression and model composite endpoints. The flexibility and diverse functionality of these models to handle the challenges of clinical data, make probabilistic predictions for individual patients, and accurately predict disease progression means that there are clear applications for clinical trials and precision medicine.

The approach to simulating disease progression that we describe here can be easily extended to other diseases. Widespread application of generative models to clinical data could produce synthetic datasets with lower privacy concerns than real medical data^10^, or could be used to run simulated clinical trials to optimize study design or as synthetic control arms. In certain disease areas, tools that use simulations to forecast risks for specific individuals could help doctors choose the right treatments for their patients. Currently, progress towards these goals is slowed by the limited availability of high quality longitudinal health datasets and the limited ability of current machine learning methods to produce insights from these datasets.

## Methods

### Data Processing

Our statistical model was trained and tested on data extracted from the Coalition Against Major Diseases (CAMD) Online Data Repository for AD (CODR-AD)^34,35^. The development and composition of this database have been previously described in detail^35^. The CAMD database contains 6955 patients from the placebo arms of 28 clinical trials on MCI and AD. These trials have varying duration, visit frequency, and inclusion criteria; nearly all patients have no data beyond approximately 18 months. We chose a 3-month spacing between time points based on the visit frequency of the bulk of long-lasting patients to ensure that most patients had no gaps in their data. The falloff in patient data after the 18-month time point led us to select that as the final time point. Therefore, patient trajectories are represented by 7 time points (0, 3, 6, 9, 12, 15, and 18 months).

Data in the CAMD database is stored in the CDISC format^49,50^. The covariates used in our statistical model of AD progression originate from tables in the database on demographics, disposition events, laboratory results, medical histories, questionnaires, subject characteristics, subject visits, and vital signs. We designated some variables, such as height, as static. Multiple values for any of the static variables were averaged to produce a single estimate. Time-dependent variables were bucketed into 90-day windows centered on each time point. Multiple entries in any window were averaged, or extremal values were taken as appropriate. Any data with units (such as laboratory tests) were converted to a common unit for each test for all patients (e.g., g/L for triglycerides). Results for both the ADAS-Cog and MMSE tests were available for many patients to the level of individual components. Individual question data were available for some patients, which we aggregated into component scores. A final processing step converted data into numerical values more suitable for statistical modeling. Categorical variables were one-hot encoded and positive continuous variables were log-transformed and standardized. All variables were transformed back to canonical form before analysis.

Our statistical model can perform imputation of missing data during training. However, using covariates that are missing in a large fraction of patients would lead to poor performance. Therefore, we chose 44 variables that were observed in a reasonably large fraction of patients. Table 1 describe each of the variables included in our analysis. Because we are interested in modeling AD progression, we focused on patients in the CAMD database with long trajectories. This led us to select the 1909 patients from CAMD that have a valid ADAS-Cog score (i.e., data is not missing for any of the 11 components) for either of the 15-month or 18-month time points.

One feature of the real patient data that complicates the comparison in Fig. 4 is the presence of missing data. To handle missing data, we mean impute each missing variable. Because the synthetic data has no missing entries, this would create a significant difference between real and synthetic data and a classifier would be able to distinguish them based solely on the missing data. However, since there is a one-to-one correspondence between real and synthetic patients, we assign the mean imputed entries to the corresponding entries in the synthetic data. This removes the ability of the logistic regression to distinguish between the two groups based on the missing-ness of data. We note that as a natural consequence, higher proportions of missing data limit the classification ability of the logistic regression.

## Supplementary information

Supplementary Information

Dataset 1

## Data Availability

Data used in the preparation of this article were obtained from the Coalition Against Major Diseases (CAMD) database. In 2008, Critical Path Institute, in collaboration with the Engelberg Center for Health Care Reform at the Brookings Institution, formed the Coalition Against Major Diseases (CAMD). The Coalition brings together patient groups, biopharmaceutical companies, and scientists from academia, the U.S. Food and Drug Administration (FDA), the European Medicines Agency (EMA), the National Institute of Neurological Disorders and Stroke (NINDS), and the National Institute on Aging (NIA). The Coalition Against Major Diseases (CAMD) includes over 200 scientists from member and non-member organizations. The data available in the CAMD database has been volunteered by CAMD member companies and non-member organizations.
