# Supplementary material for: Machine learning for comprehensive forecasting of Alzheimer’s Disease progression
Source: Sci Rep. 2019 Sep 20;9:13622. doi: 10.1038/s41598-019-49656-2 (PMC6754403; doi:10.1038/s41598-019-49656-2)
Supplement: Supplementary file 1 — Supplementary Information [file 41598_2019_49656_MOESM1_ESM.pdf]

Supporting Information to "Machine learning for comprehensive forecasting of Alzheimer's Disease progression"

Charles K. Fisher,* Aaron M. Smith, and Jonathan R. Walsh

Unlearn.AI, Inc., 650 California St, San Francisco, CA 94108

for the Coalition Against Major Diseases†

* drckf@unlearn.ai; authors listed alphabetically.

† Data used in the preparation of this article were obtained from the Coalition Against Major Diseases database (CAMD). As such, the investigators within CAMD contributed to the design and implementation of the CAMD database and/or provided data, but did not participate in the analysis of the data or the writing of this report.

I. SUPPORTING INFORMATION

A. Data Processing

The CAMD database stores data using CDISC standards, specifically the Study Data Tabulation Model (SDTM), which defines a common schema for clinical trial data and is the required standard for clinical data submissions to the United States Food and Drug Administration (FDA). In this format the data is already highly structured; therefore it is possible to develop data processing pipelines that can apply to SDTM data in general and not simply the particular database used here. We describe the general architecture of our data processing pipeline and the CAMD-specific processing used.

The goal of our processing pipeline is to arrive at data that may be directly used by machine learning algorithms to build patient-level models. This means:

- Data must be numerically formatted, such as numeric values, ordinal values for scores, and one-hot encoding for categorical variables. For text or image data, this may involve feature extraction, e.g. through a word2vec model or an autoencoder.

- Data must be patient-specific, and can extend over time in regular intervals. For example, if we have cholesterol measurements for a given patient at 1, 2, 5, and 12 months, but are modeling the population at 3-month intervals, then we may average the 1- and 2-month time point values and will have a missing entry between the 5- and 12-month values.

Data arrives in Comma Separated Value (CSV) formatted text files, with abbreviation encodings for file and variable names. Many of these abbreviation are generic to SDTM and some apply specifically to disease areas. A translation table, such as one provided by CAMD, may be used to automatically convert abbreviations to human-readable names. Using this translation and simple type inference on variables, the data is ingested into a SQL database via a simple script. We label this data in this form as the *raw database*.

The main component of the processing pipeline extracts data appropriate for training and evaluating machine learning algorithms. This is done on a per-variable basis, meaning the primary functions in the pipeline produce data for only a single variable; this processing is then repeated over all variables of interest. Processed data is stored in the *processed*

database and may directly be used to construct datasets for machine learning. The steps in the processing are:

- Declare which columns and tables from the raw database will be used to produce the data for a given variable.
- Declare a processing function to convert this data into the appropriate form.
- Declare a location in the processed database where the data will be stored.
- Query the raw database for the data, apply the processing function, and store the result in the processed database.

The processing functions may be common, such as one-hot encoding categorical labels, or they may be custom, such as standardizing units for a particular laboratory measurement. Such custom functions form the bulk of database-specific code that must be written. All of the above processing steps can easily be encoded in configuration files, meaning the process of preparing data for machine learning is simple and repeatable.

Finally, datasets may be constructed from the processed database by merely specifying which variables are to be used. This step is also performed via a configuration file. Additional filtering of patients, e.g. by requiring they have data present for a certain number of time points, is straightforward to apply.

We have developed a `python` library to process data as described above. This library fully handles the interface with the SQL database, has common data conversion functions, and provides utilities to provide summary statistics and type inference for variables in a dataset. For any specific project, such as the CAMD database, most of the processing is set up by writing `YAML` configuration files that are simple, human-readable, and easily verified. The remainder involves writing custom processing functions in `python` for specific variables. This setup makes it straightforward to apply our machine learning models to other clinical data modeling problems.

1. Variables Used in Training

Variables relevant to modeling AD progression were extracted from the CAMD database using the method described above, and 44 variables without substantial missing data were

Category	Name	Units	Notes
ADAS	Commands	counts	0 – 5 ordinal range
ADAS	Comprehension	counts	0 – 5 ordinal range
ADAS	Construction	counts	0 – 5 ordinal range
ADAS	Delayed Word Recall	counts	0 – 10 ordinal range
ADAS	Ideational	counts	0 – 5 ordinal range
ADAS	Instructions	counts	0 – 5 ordinal range
ADAS	Naming	counts	0 – 5 ordinal range
ADAS	Orientation	counts	0 – 8 ordinal range
ADAS	Spoken Language	counts	0 – 5 ordinal range
ADAS	Word Finding	counts	0 – 5 ordinal range
ADAS	Word Recall	counts	0 – 10 ordinal range
ADAS	Word Recognition	counts	0 – 12 ordinal range
MMSE	Attention and Calculation	counts	0 – 5 ordinal range
MMSE	Language	counts	0 – 9 ordinal range
MMSE	Orientation	counts	0 – 10 ordinal range
MMSE	Recall	counts	0 – 3 ordinal range
MMSE	Registration	counts	0 – 3 ordinal range

TABLE I. Cognitive variables included in the model.

identified and used for the model. Tables I and II lists all variables, their units, and specific processing considerations for each. Each laboratory test variable is converted to the units given, and all transformations applied to train the models are inverted for analysis. Ordinal variables are rescaled to maximum value 1 during training, and unscaled for analysis. A supplemental CSV file is provided containing summary statistics (mean, standard deviation, and fraction missing) for each variable at each time point, extending the baseline information provided in Table I in the main text.

Of the 6945 patients in the CAMD database, very few have data after approximately 18 months from baseline. A 3-month (90-day) interval was a suitable interval such that most patients have data at every time point; shorter intervals yielded groups of patients without

data at some time points. Therefore, we chose to represent all temporal variables in 3-month intervals from 0 (baseline) to 18 months, giving 7 available time points for each patient.

2. *Patients Used in Training*

To model progression, we are most interested in patients with longer trajectories. Therefore, we selected the 1909 patients that have data at the 15- or 18-month time points. All evaluation, including CRBMs and supervised models, was performed using 5-fold cross validation (CV) on this dataset. Each fold has a distinct division of these 1909 patients into training, validation, and testing groups. To construct these CV datasets, we randomly divided the 1909 subjects into equal (up to one patient) groups each containing 20% of the data. For each fold, the 20% of the data is used as the test dataset, and the remaining data is divided into a training set (with 75% of all samples) and a validation set (the remaining 5% of all samples). In each fold, models are trained on the training set, hyperparameters validated on the validation set, and then evaluated on the testing set. All results shown in this paper are from evaluation on these test sets or other completely out-of-sample data.

B. Motivation for CRBMs

Boltzmann machines are a well-known, standard machine learning algorithm for modeling relationships between data. They provide several features critical to modeling clinical data not found in most machine learning models:

- They can easily model multimodal data. Different neuron types may be used to model continuous numeric, ordinal, Bernoulli, or categorical data.
- They allow for conditional and generative sampling. If some clinical data is known for a patient, it can be used to predict unknown data for that patient. For example, an initial population of an AD clinical trial may be defined in terms of standard inclusion criteria, such as age, sex ratio, and ADAS-Cog scores, and the remaining baseline data and any future data may be predicted.
- As a consequence, they naturally handle missing data. The model itself may be used to impute missing values from the learned joint probability distribution of the data.

This may be done *during training*, meaning missing data can be directly fed into the model.

- They are stochastic, meaning data may be sampled. Stochastic models naturally provide an estimate of their uncertainty through this sampling. For clinical data, the consequence is that the model returns both a prediction and an uncertainty for any clinical variable being predicted.

Conditional Restricted Boltzmann Machines [1–4] provide a way to model time series data using the natural capabilities of Boltzmann machines. Our CRBM contains the visible units for multiple time points, with a standard hidden layer. The visible units are organized as:

$$\mathbf{v}_{\text{CRBM}} = \mathbf{v}_{\text{static}} \oplus \mathbf{v}_t \oplus \cdots \oplus \mathbf{v}_{t+k} \,, \tag{1}$$

where k is the time lag of the model and $\oplus$ signifies concatenation. In our model, we use $k = 1$, so that two time points are learned simultaneously. The static units are only used once over all time points, as they are constant over all times. The model learns the complete joint probability distribution between all $k+1$ adjacent time points simultaneously, $p(\mathbf{v}_{\text{static}}, \mathbf{v}_t, \mathbf{v}_{t+1}, \ldots, \mathbf{v}_{t+k})$. That means that *any* conditional sampling of the data may be performed, such as predicting the data for a time point given the previous k time points. A baseline cohort may be simulated by sampling from the model and using the first time point. This treatment of the data to allow for learning inter-dependence between time points is the only distinction of a CRBM over a standard RBM.

C. Details of the CRBM Implementation

Unlike feedforward neural networks, there is no widely adopted computational library providing a high-level interface to build and research Boltzmann machines. However, there are nascent libraries aimed at providing these tools [5]. Alternatively, the fundamental algorithms needed to represent, train, and sample from Boltzmann machines may be built from commonly used tensor manipulation and machine learning libraries. Here we describe the ingredients in the implementation of CRBMs used in this work.

A CRBM is a type of Restricted Boltzmann Machine (RBM), where the organization of the visible units is particular to the time-dependence of the data. That is, without particular

temporal labels on the visible units of the CRBM, the model is simply an RBM. CRBMs can be trained the same way as RBMs, except that the temporal nature of the data allows for a different organization of the data used to train the model; the following subsection describes the particulars of training CRBMs in more detail.

An RBM is a Boltzmann Machine where the connections in the model are restricted to only being inter-layer, meaning visible units are connected to hidden units but there is no direct connection between visible units or between hidden units. The model is represented in terms of a set of parameters, namely those in Equation 1 in the main text. Recalling that equation,

$$p(\mathbf{v}, \mathbf{h}) = Z^{-1} \exp\left(\sum_j a_j(v_j) + \sum_\mu b_\mu(h_\mu) + \sum_{j\mu} W_{j\mu} \frac{v_j}{\sigma_j^2} \frac{h_\mu}{\epsilon_\mu^2} \right), \tag{2}$$

Each unit of the visible and hidden layers has bias parameters determined by the choice of functions a_j and b_μ, as well as scale parameters σ_j and ϵ_μ. The connection between the layers is parameterized by the weights $W_{j\nu}$.

Simulations with an RBM are performed via sampling. For any particular data type, Equation 2 specifies how to sample values of the visible or hidden units, where conditioning on particular unit values may occur. However, for any unit, we need to know the constraints of the data type for that unit; for example, if the unit represents a Bernoulli variable that only takes on values 0 and 1, then sampling should only return those values. These constraints are encoded in sampling functions that define conditional probability distributions for each data type (and consequently allow for sampling for each unit). Because sampling from Equation 2 directly is computationally infeasible, sampling is performed via Markov Chain Monte Carlo (MCMC), described well in [6]. In MCMC, all visible units are sampled conditioned upon all hidden units, and then vice versa, with this procedure repeating many times to sample from the approximate joint probability distribution defined in Equation 2.

Our implementation of RBMs uses the above methods to parameterize and sample from models. Training is performed by using the analytically computed gradients from each parameter under the objective function to determine updates to the model. Like the conditional probabilities used in sampling, these gradients are expressed as functions that are called as necessary to numerically compute values. Training uses the same essential sampling methods described above, with the samples from the data and the model both used to compute gradient updates as described in [2].

D. Details of Training

The CRBM is trained on the data from adjacent pairs of time points. If $\mathbf{x}_t$ is the vector of time-dependent variables for a patient at time t and $\mathbf{x}_{\text{static}}$ is the vector of static variables for the same patient, then the visible units used to train the CRBM are $\mathbf{v} = \{\mathbf{x}_{t+1}, \mathbf{x}_t, \mathbf{x}_{\text{static}}\}$, a concatenation of the data from the adjacent time points t and $t+1$ with the static variables represented only once.

When training the CRBM, the data for each patient in the training and validation groups are reorganized into all adjacent pairs of frames. Since each patient has data for 7 time points, they contribute 6 pairs of time points (which we will call samples). Inside of each group samples are all shuffled so that minibatches contain a mixture of patients and times.

The CRBM has a single hidden layer of 50 ReLU units, and is trained using the methods described in [7]. The objective function $\mathcal{C}$ is a linear combination of log-likelihood $\mathcal{L}$ and adversarial $\mathcal{A}$ objectives,

$$\mathcal{C} = -\gamma\mathcal{L} - (1-\gamma)\mathcal{A}\,, \tag{3}$$

where γ is a parameter weighing the relative size of the two objectives. Parameters used to train the CRBM are listed in Table III. The training setup is similar to those used in [7]; here we use a random forest classifier for the adversary. Temperature-driven sampling, where the temperature is sampled from an autocorrelated Gamma distribution, is not used, though the model performance is not especially sensitive to this choice.

Notable dynamics were observed during the training process. Within 100 epochs, metrics monitored during training such as KL divergence and reverse KL divergence achieve values close to their final values. Sampling from the model at this stage, the model has relatively poorer performance for patients with extremal ADAS-Cog scores than those near the mode. Most importantly, during the early stages of training the model has a strong regression to the mean effect for ADAS-Cog score outliers, where the model predicts patients with a low score progress rapidly and those with a high score *improve* – the opposite of what is observed in the data. Continuing training allows the model to unlearn this behavior and correctly learn a progression from the mean, where higher scoring individuals progress more rapidly. We expect that this feature of training, where it takes a longer time to effectively learn the behavior of outliers, is common.

E. Details of Progression Predictions

This subsection gives details on the modeling for Figure 5B. A linear regression, random forest regression, and neural network were trained to predict the ADAS-Cog score change from the baseline patient data at a single given readout time. The CRBMs are also used to predict the same score change. The performance of all the algorithms are very similar; it is likely additional patients or additional data predictive of patient progression would be needed to substantially improve the performance of the models. For example, we found that the addition of the ApoE $\epsilon 4$ allele count to the baseline variables decreases the RMS error by 5–10%. This section gives details on the training of the supervised models and the evaluation of all models.

The supervised models are trained from the baseline time point data to predict the ADAS-Cog score change from baseline to readout. All possible time points (3, 6, 9, 12, 15, and 18 months) are used as readout times, and separate supervised models must be trained for each readout time. The same CRBM model may be used for all readout times.

However, there is missing data for many patients. We exclude any patients that having any missing ADAS-Cog components at baseline or readout, ensuring that valid labels can be defined. For the supervised models, we mean impute other missing baseline variables from the training data. After this screening, the number of training patients for the supervised algorithms and testing patients for all algorithms as a function of readout time is given in Table IV.

Both the CRBMs and supervised models are evaluated using 5-fold cross validation. This means that for each fold, the models are evaluated only on the test data for that fold. For the CRBMs, this data is out-of-sample from the training set (as it is for all other figures in this paper). The supervised models are trained on the training data for each fold, and cross validation is used within each fold to select the optimal hyperparameter and further avoid overfitting. All algorithms are evaluated on the same test data for each fold; Figure 5B shows the mean RMS error over all CV folds along with the uncertainty (the standard deviation over the folds). Table V gives the architectures and hyperparameters for each of the supervised algorithms. Note that for the supervised algorithms, a new model was trained for every time point, while the CRBM is the same model over all time points.

Once trained, the supervised algorithms are used to predict the ADAS-Cog score change

for the test data for each CV fold, and a root mean square (RMS) error over the test set is computed. This is done for each readout time. The predictions for the CRBM (over all readout times) are obtained by repeatedly simulating patient trajectories from the baseline time point. For each simulation, the ADAS-Cog score change is recorded, yielding a distribution of score changes *for each patient* that represents the probabilistic distribution of predictions made by the CRBM. The mean of this distribution is the prediction of the CRBM for the given patient. The RMS error of these predictions is computed, as it was for the supervised models. These results make up the data shown in Figure 5B.

F. Details of Trajectory Progression Predictions

This subsection gives details on the modeling for Figures 3 and 4. The approach is the natural extension of the methodology described in the previous subsection.

On each CV fold, a random forest is trained to predict a single time-dependent variable at a single readout time, meaning over all 35 time-dependent variables and all 6 possible readout times, 210 different random forest models are trained. The input data are the baseline variables, with mean imputation used in the case of missing data. Samples where either the baseline value or the readout value (the label) are missing are excluded. The root mean square (RMS) error is computed over the test data, again only using samples where the variable being modeled is present at both baseline and the readout time. For the CRBM, predictions are made by repeatedly simulating patients conditioned on their baseline data, and taking the mean for each patient as the CRBM prediction. The RMS error can then be computed using the same test samples on which the random forest models were evaluated.

It is helpful to normalize these errors by the standard deviation of the value to be predicted. An error ratio of 1 implies that the prediction is no better than predicting the mean of the test data, and an error ratio well below 1 implies that the prediction is highly precise at a per-patient level.

G. Additional Results

There are many ways to study the performance of an unsupervised model, so we take the opportunity to present additional results that provide more insight into the CRBM.

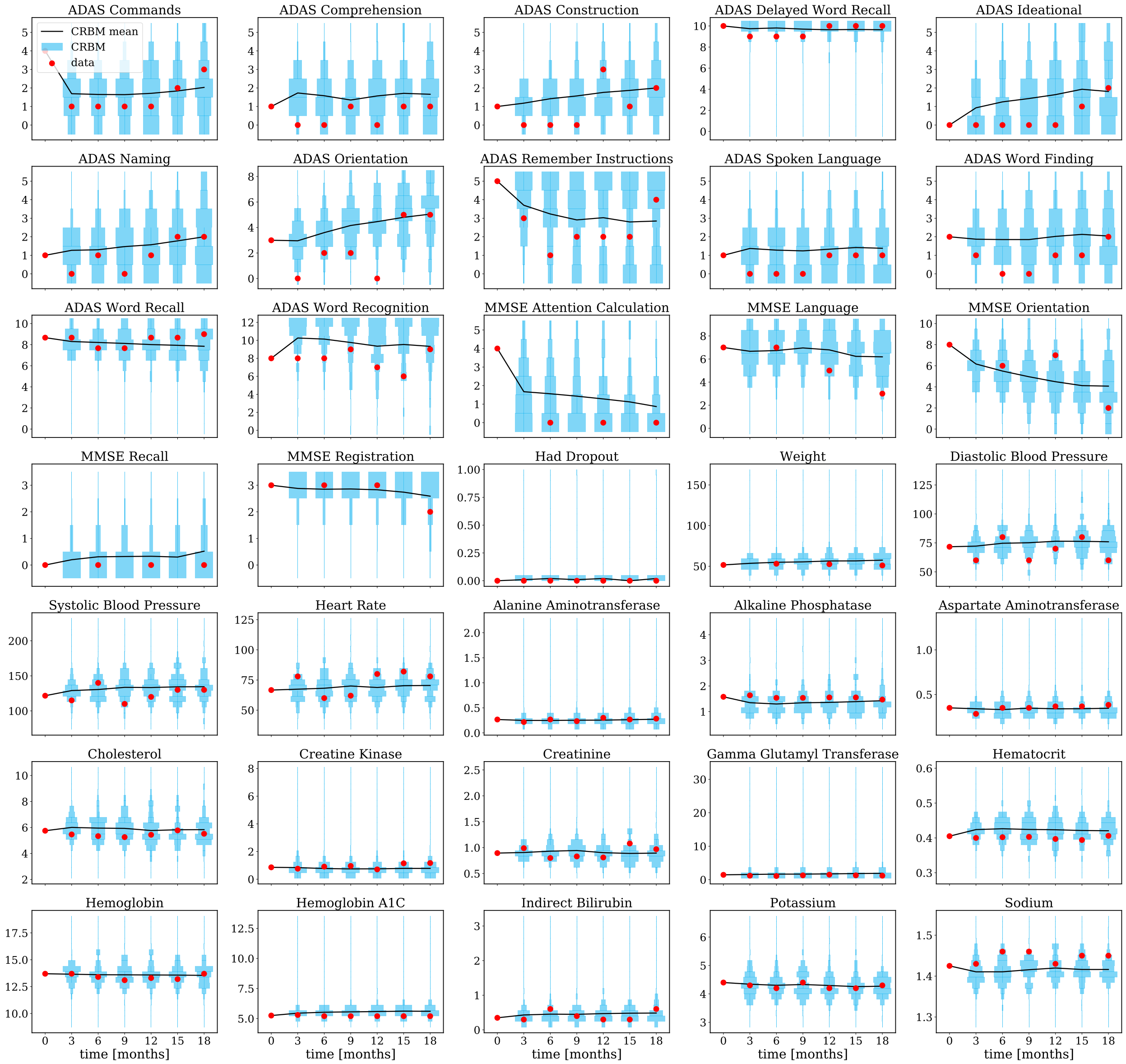

FIG. 1. **Stochastic simulations enable individual assessments of risk**. Violin plots display the stochastic evolution of an example patient over 18 months. The width of the blue bars represents the probability computed using simulations from the CRBM (the one corresponding to the CV fold where the example patient is part of the test data), and the mean CRBM prediction is shown as the black line. The red dots show the actual observed values from the chosen patient. The CRBM was initialized with the observed values at baseline ($t = 0$). Then, we repeatedly simulated 18-month trajectories and created histograms of each variable at every time point. The model predicts trends and imputes values when observations are missing for the patient (e.g., MMSE components at some time points). Units for these data are given in Table II.

As discussed in the main text, the CRBM is able to provide accurate estimates of its uncertainty while forecasting the progression of individual patients. In Figure 1, we take an example patient from one CV fold and plot the values for each time-dependent variable for that patient. We condition on the baseline patient data and repeatedly simulate 18-month trajectories. These simulations form a distribution of predictions for each variable, which we show along with the mean. The CRBM captures the trends of the data and provides an uncertainty estimate by virtue of the simulation.

The marginal distributions of the CRBM are plotted in Figures 2, 3, and 4. In Figure 2, the CRBM models are used to freely generate simulated patients (without conditioning on any data) and the distribution for each variable is plotted against the data. In Figure 3 and 4, the marginal distributions for each variable at each time point are plotted, conditioning the CRBM models on the baseline patient data in the test group for each CV fold. In all figures, missing values for a particular variable are not used when computing the data distribution (these values are dropped), while imputation with the CRBM models is performed. The data shown includes all CV models together, combining data from each fold to form higher-statistics distributions. Additionally, in Figure 5, the first two moments (the mean and standard deviation) of the conditional marginal distributions shown in Figure 3 and 4 are compared. A t-test is used to quantify the agreement of the means, and Levene's test is used to quantify the agreement of the standard deviations [8]. Excellent agreement is observed, indicating these moments of the marginal distributions are accurate.

Figure 2 in the main text shows that the CRBM captures the correlations and autocorrelations when the model is conditioned upon baseline data. In Figure 6, we show the same is true when freely generating virtual patients. For each CV fold, we simulate 18-month trajectories. For the data and these simulated patients, we compute the correlations and autocorrelations between variables. This figure is the companion to Figure 2 in the main text, where in that figure we conditioned synthetic patients on baseline data while in Figure 6 we generated synthetic patients without conditioning. Because the synthetic patients in this case are more closely related to the data, the correlation coefficients from the CRBMs are closer to the data than in this case, as evidenced by the R^2 values of the correlation and autocorrelation relationships.

Similarly, Figure 7 provides a complementary figure to Figure 5C in the main text. In that figure, we used the model to generate cohorts of fast and slow progressing patients with

MCI and an ADAS-Cog score of 10. In this case we adopt the same methodology, but with.a different initial condition: we use AD patients with an initial ADAS-Cog score of 20.

To further validate of the performance of the CRBMs, in Figure 8 we compare data from the control arm of a 12-month clinical trial in the CODR-AD database to each of the 5 CRBM models trained during CV. The CRBMs agree reasonably well with the data, predicting the progression more accurately for the later time points in the trial, especially the 12-month time point. The early-time progression predicted by the CRBMs is higher than the data, likely due to the placebo effect [9]. To perform this comparison, we conditioned the CRBMs on baseline data of subjects in the trial and simulated 12-month trajectories for each patient. The values shown at each time point only include data from the subjects who remained in the trial with a valid ADAS-Cog score; any subject who drops out is not included and is also dropped from the CRBM simulations at the corresponding time point.

In Figure 9, we show a statistical measure of the ADAS-Cog progression predictions from the CRBM, using data generated for Figure 5B in the main text. The CRBM predictions form a distribution, and from this distribution we calculate the mean and standard deviation, μ and σ. These, together with the observed progression value in the data, ν, can form a normalized score, $z = (\mu - \nu)/\sigma$. The distribution of z-scores should be approximately normal when sampling from the true data population, which is the goal of the simulation. One can see that the distribution of z-score values is close to normal, and the confidence of the CRBM (the standard deviation of its prediction) is uncorrelated with the z-score value.

Like Figure 1, particular example patients are of interest. In Figure 10 we plot the distribution of ADAS-Cog progression predictions from the CRBM for three example patients, showing the CRBM mean prediction (the mean value of the distribution) against the true progression in the data. The examples highlight the range of predictions from the CRBM; it is more confident for some subjects, having a distribution with lower standard deviation, and less confident for other subjects. In Figure 11, we show example ADAS component progression for three example patients. The CRBM is conditioned on the baseline data for these patients and repeated simulations are made of each patient's trajectory. Each ADAS-Cog component is averaged and plotted in the figure. This highlights the fact that the ADAS-Cog is a multidimensional endpoint, showing the different ways in which patients progress.

Finally, in Figure 12 we show the performance of the CRBM models against a "global"

random forest that predicts all variables at a single time point. This model is described above, and the figure is a direct companion to Figure 3 in the main text. Such a model would be able to accurately capture the covariance between variables, but lacks the precision necessary to be useful – it rarely performs better than the CRBM.

---

[1] D. H. Ackley, G. E. Hinton, and T. J. Sejnowski, Cognitive science **9**, 147 (1985).

[2] G. Hinton, Momentum **9**, 926 (2010).

[3] G. W. Taylor, G. E. Hinton, and S. T. Roweis, in *Advances in neural information processing systems* (2007) pp. 1345–1352.

[4] V. Mnih, H. Larochelle, and G. E. Hinton, in *Proceedings of the Twenty-Seventh Conference on Uncertainty in Artificial Intelligence* (AUAI Press, 2011) pp. 514–522.

[5] S. Lenz, M. Hess, and H. Binder, bioRxiv (2019), 10.1101/578252, https://www.biorxiv.org/content/early/2019/03/20/578252.full.pdf.

[6] T. Tieleman, in *Proceedings of the 25th international conference on Machine learning* (ACM, 2008) pp. 1064–1071.

[7] C. K. Fisher, A. M. Smith, and J. R. Walsh, arXiv preprint arXiv:1804.08682 (2018).

[8] H. Levene, Contributions to Probability and Statistics: Essays in Honor of Harold Hotelling , 278 (1960).

[9] K. Ito, B. Corrigan, K. Romero, R. Anziano, J. Neville, D. Stephenson, and R. Lalonde, Journal of Alzheimer's Disease **37**, 173 (2013).

**Category**	**Name**	**Units**	**Notes**
Laboratory	Alanine aminotransferase	μkat/l	log-standarized for training
Laboratory	Alkaline phosphatase	μkat/l	log-standarized for training
Laboratory	Aspartate aminotransferase	μkat/l	log-standarized for training
Laboratory	Cholesterol	mmol/l	log-standarized for training
Laboratory	Creatine kinase	iu/cl	log-standarized for training
Laboratory	Creatinine	mg/dl	log-standarized for training
Laboratory	Gamma glutamyl transferase	iu/dl	log-standarized for training
Laboratory	Hematocrit	counts	log-standarized for training
Laboratory	Hemoglobin	g/dl	log-standarized for training
Laboratory	Hemoglobin a1c	%	log-standarized for training
Laboratory	Indirect bilirubin	mg/dl	log-standarized for training
Laboratory	Potassium	mmol/l	log-standarized for training
Laboratory	Sodium	mmol/cl	log-standarized for training
Laboratory	Triglycerides	g/l	log-standarized for training
Clinical	Blood pressure (diastolic)	mmHg	log-standarized for training
Clinical	Blood pressure (systolic)	mmHg	log-standarized for training
Clinical	Heart rate	bpm	log-standarized for training
Clinical	Weight	kg	log-standarized for training
Clinical	Dropout	-	1 for dropout before the next time
Background	Age at baseline	Years	'>89' $\rightarrow$ 90, log-std^{ized} for training
Background	Geographic region	-	1-hot, 7 labels built from country
Background	Initial diagnosis (AD or MCI)	-	Bernoulli
Background	Past cardiovascular event	-	Bernoulli
Background	ApoE $\epsilon 4$ allele count	counts	0, 1, or 2
Background	Race	-	1-hot, 6 labels
Background	Sex	-	Bernoulli, 1 if female
Background	Height	cm	log-standarized for training

TABLE II. Laboratory, clinical, and background variables included in the model.

**Hyperparameter**	**Value / Notes**
number of epochs	2000
batch size	100
training/validation fractions	93.75% / 6.25%
learning rate	0.005 initial; 0 final; linear decay
optimizer	ADAM, beta $(0.9, 0.999)$
Monte Carlo steps (sampling)	50
Monte Carlo steps (imputation in training)	2
driven sampling β	0
likelihood weight γ (Equation 3)	0.3
adversary	random forest, 5 trees with max depth 5
weight penalty	ℓ_2 penalty with coefficient 10^{-1}

TABLE III. Hyperparameters used to train the CRBM.

**Time [months]**	**Training Patients**	**Testing Patients**
3	1506 / 1508 / 1505 / 1505 / 1508	377 / 375 / 378 / 378 / 375
6	1504 / 1501 / 1502 / 1501 / 1504	374 / 377 / 376 / 377 / 374
9	1492 / 1489 / 1488 / 1489 / 1494	371 / 374 / 375 / 374 / 369
12	1498 / 1485 / 1493 / 1488 / 1488	365 / 378 / 370 / 375 / 375
15	1506 / 1503 / 1498 / 1500 / 1505	372 / 375 / 380 / 378 / 373
18	1385 / 1380 / 1384 / 1386 / 1393	347 / 352 / 348 / 346 / 339

TABLE IV. Number of training and testing patients used to predict ADAS-Cog score progression as a function of readout time for each CV fold.

Model	Architecture	Hyperparameters
Linear Regression	ridge (L_2 regularization)	$\alpha \in \{10^k\}_{k=-3}^{2}$
Random Forest	100 trees	max depth $\in \{2^k\}_{k=2}^{6}$
Neural Network	2 hidden layers (30, 10) units<br>ReLU activations	ADAM learning rate = 0.02<br>batch size = 25<br>20 epochs

TABLE V. Supervised models used to predict ADAS-Cog score progression.

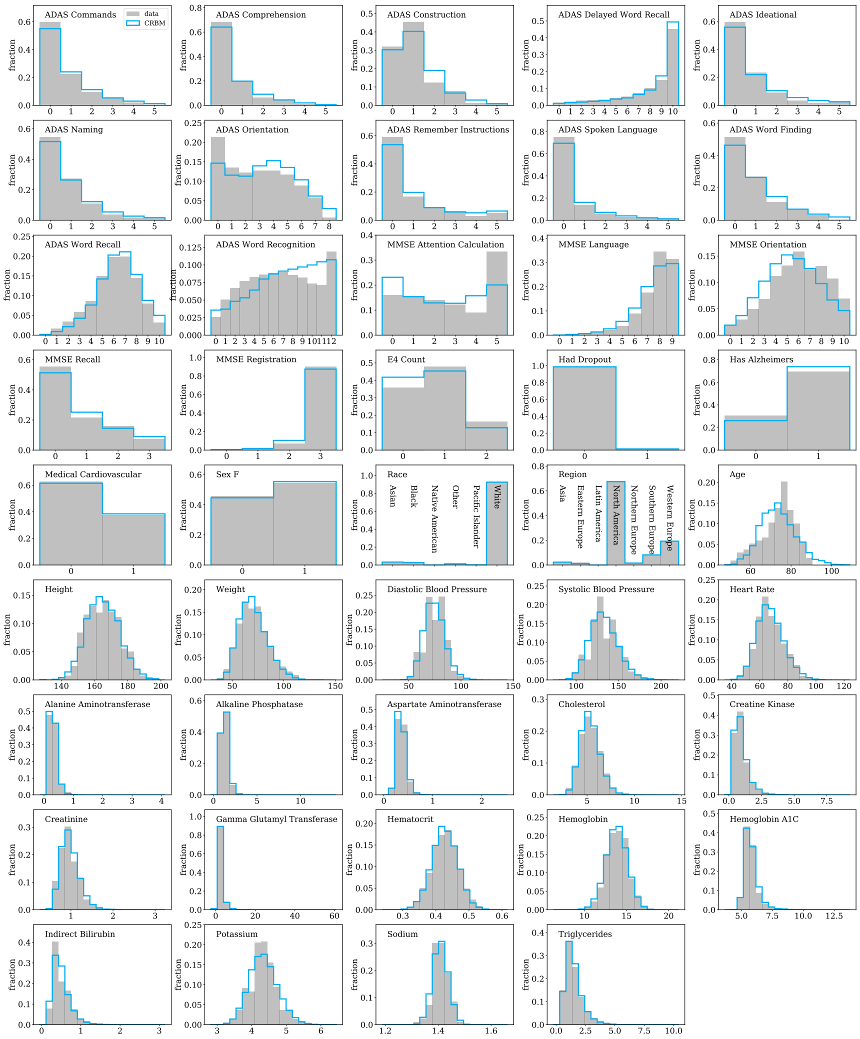

FIG. 2. **Marginal distributions in the generative mode for all variables.** For each fold, the CRBM is used to generate 18-month patient trajectories with the same number of virtual patients as the number of patients in the test group for that fold. The marginal distributions for the test group patients and the CRBMs are shown for all variables, combined over all folds. For each variable, patients with missing data are not included in the data distribution (no imputation is performed), though for the CRBM imputation is performed and all patients are included.

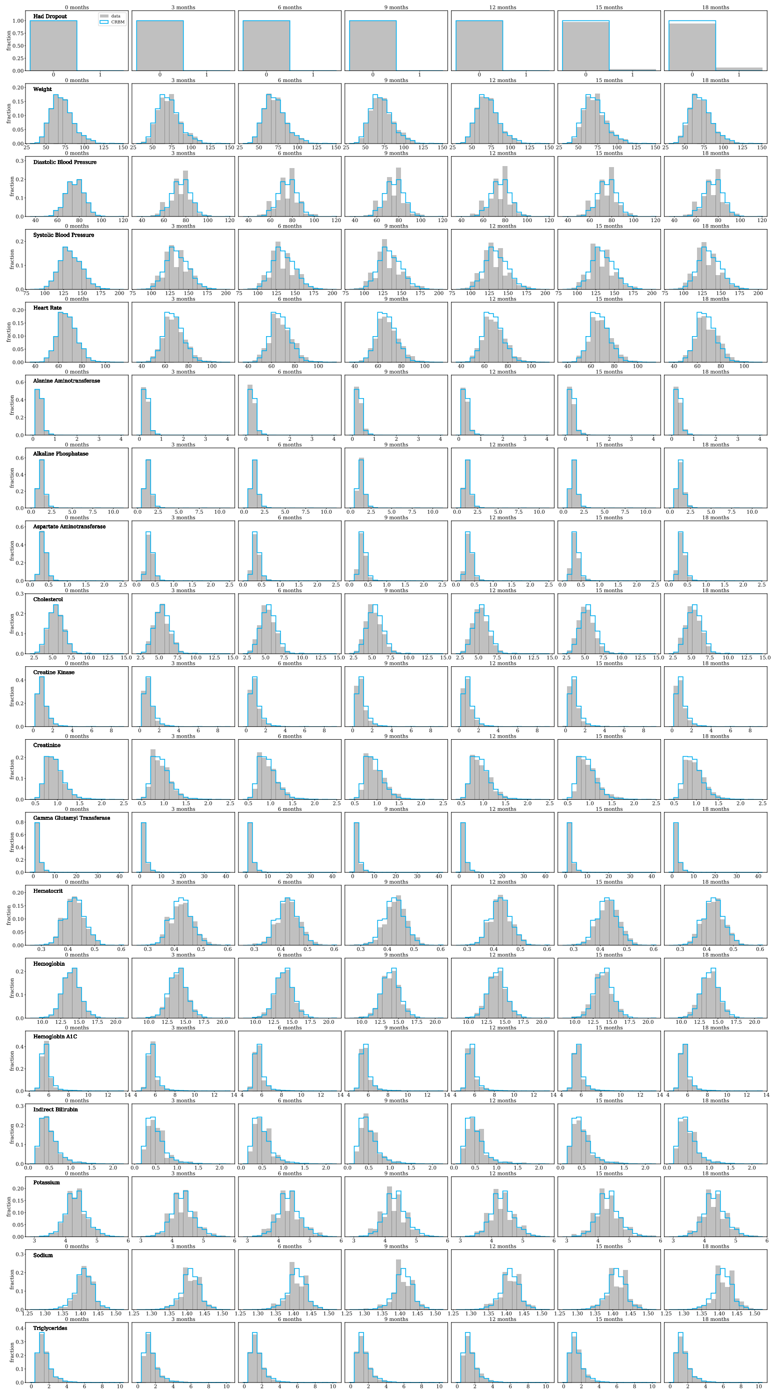

FIG. 3. **Marginal distributions conditioned on baseline for clinical variables.** For each fold, the CRBM is conditioned on the patient data at baseline, and one trajectory is simulated for each patient in the test group for that fold. The marginal distributions for the patients and the CRBM are shown for clinical variables, combined over all folds. Because the CRBM is conditioned on the data at baseline, the 0-month distributions always match except when the data is missing values and the model performs imputation. For each variable at each time point, patients with missing data are not included in the data distribution (no imputation is performed), though for the CRBM imputation is performed and all patients are included.

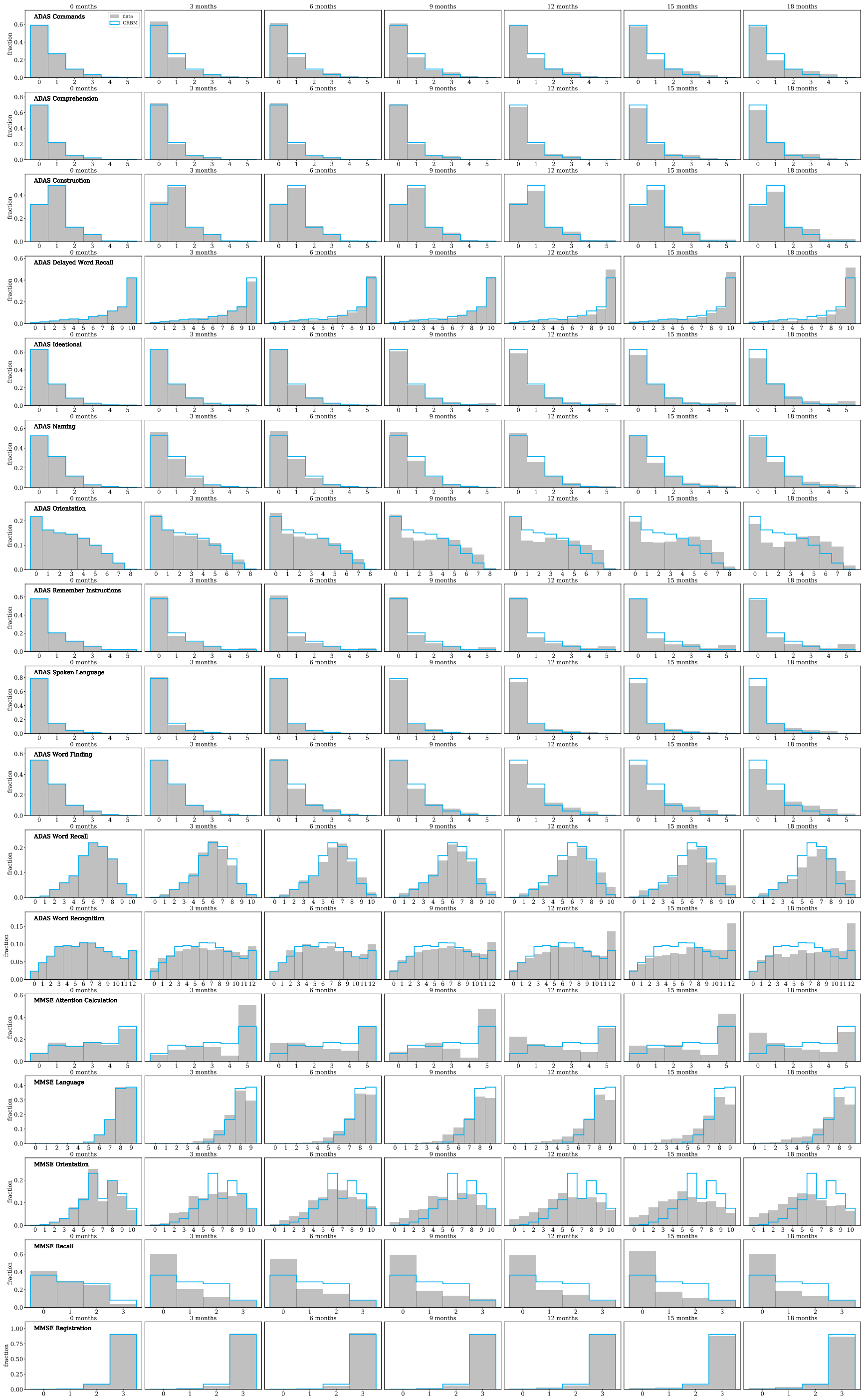

FIG. 4. **Marginal distributions conditioned on baseline for cognitive variables.** For each fold, the CRBM is conditioned on the patient data at baseline, and one trajectory is simulated for each patient in the test group for that fold. The marginal distributions for the patients and the CRBM are shown for cognitive variables, combined over all folds. Because the CRBM is conditioned on the data at baseline, the 0-month distributions always match except when the data is missing values and the model performs imputation. For each variable at each time point, patients with missing data are not included in the data distribution (no imputation is performed), though for the CRBM imputation is performed and all patients are included.

mean, data	mean, CRBM	**p-value (mean)**	
std, data	std, CRBM	**p-value (std)**	

Variable	3 months	6 months	9 months	12 months	15 months	18 months
ADAS Commands	0.61 0.64 **0.29**	0.70 0.70 **0.07**	0.75 0.80 **0.50**	0.78 0.73 **0.50**	0.87 0.82 **0.42**	0.85 0.86 **0.05**
	0.96 0.92 **0.71**	0.98 0.98 **0.93**	1.09 1.09 **0.50**	1.13 1.05 **0.50**	1.18 1.15 **0.58**	1.22 1.20 **0.95**
ADAS Comprehension	0.45 0.44 **0.17**	0.49 0.55 **0.63**	0.55 0.62 **0.70**	0.65 0.63 **0.23**	0.71 0.67 **0.40**	0.73 0.72 **0.15**
	0.82 0.88 **0.83**	0.88 0.92 **0.38**	0.96 0.92 **0.30**	1.06 0.99 **0.77**	1.10 1.09 **0.60**	1.11 1.11 **0.85**
ADAS Construction	0.96 1.02 **0.65**	0.98 1.06 **0.70**	1.0 1.1 **0.73**	1.1 1.1 **0.45**	1.2 1.3 **0.84**	1.1 1.2 **0.71**
	0.91 0.94 **0.83**	0.93 0.99 **0.36**	1.0 1.0 **0.81**	1.0 1.1 **0.89**	1.1 1.1 **0.97**	1.1 1.1 **0.67**
ADAS Delayed Word Recall	7.9 8.2 **0.95**	8.2 8.3 **0.62**	8.1 8.3 **0.61**	8.3 8.3 **0.26**	8.1 8.4 **0.73**	8.3 8.4 **0.16**
	2.6 2.3 **0.04**	2.5 2.4 **0.48**	2.6 2.6 **0.63**	2.5 2.5 **0.92**	2.7 2.5 **0.24**	2.6 2.6 **0.84**
ADAS Ideational	0.64 0.64 **0.04**	0.62 0.63 **0.17**	0.75 0.78 **0.27**	0.80 0.88 **0.60**	0.88 0.97 **0.68**	0.92 1.01 **0.63**
	1.01 1.01 **0.96**	0.96 1.06 **0.83**	1.15 1.20 **0.73**	1.23 1.29 **0.40**	1.28 1.32 **0.32**	1.28 1.32 **0.37**
ADAS Naming	0.65 0.71 **0.57**	0.69 0.76 **0.71**	0.73 0.80 **0.64**	0.85 0.91 **0.53**	0.90 0.91 **0.15**	0.89 0.91 **0.19**
	0.98 1.02 **0.43**	0.98 1.07 **0.29**	1.07 1.09 **0.36**	1.20 1.13 **0.80**	1.23 1.16 **0.85**	1.22 1.16 **0.84**
ADAS Orientation	2.6 2.7 **0.36**	2.8 2.9 **0.54**	3.0 3.1 **0.60**	3.1 3.3 **0.73**	3.3 3.5 **0.84**	3.4 3.6 **0.78**
	2.2 2.0 **0.07**	2.2 2.1 **0.50**	2.2 2.2 **0.37**	2.4 2.3 **0.26**	2.4 2.3 **0.48**	2.3 2.3 **0.97**
ADAS Remember Instructions	0.92 0.88 **0.37**	0.85 0.91 **0.47**	0.94 0.97 **0.18**	1.1 1.2 **0.65**	1.1 1.4 **0.91**	1.1 1.3 **0.74**
	1.30 1.33 **0.63**	1.26 1.38 **0.54**	1.44 1.48 **0.82**	1.5 1.6 **0.35**	1.7 1.6 **0.25**	1.6 1.7 **0.27**
ADAS Spoken Language	0.38 0.41 **0.33**	0.42 0.49 **0.64**	0.51 0.48 **0.25**	0.62 0.54 **0.68**	0.65 0.59 **0.57**	0.72 0.65 **0.59**
	0.84 0.84 **0.67**	0.91 0.92 **0.36**	1.02 1.01 **0.75**	1.09 1.06 **0.32**	1.13 1.11 **0.43**	1.26 1.13 **0.42**
ADAS Word Finding	0.73 0.83 **0.82**	0.85 0.91 **0.50**	0.89 0.93 **0.35**	0.98 1.02 **0.36**	1.1 1.1 **0.11**	1.2 1.1 **0.29**
	1.00 1.10 **0.18**	1.11 1.14 **0.94**	1.20 1.18 **0.69**	1.24 1.23 **0.52**	1.4 1.3 **0.42**	1.4 1.3 **0.10**
ADAS Word Recall	6.1 6.1 **0.45**	6.2 6.2 **0.14**	6.2 6.3 **0.64**	6.3 6.4 **0.31**	6.3 6.5 **0.82**	6.5 6.5 **0.13**
	1.8 1.8 **0.78**	1.9 1.9 **0.85**	2.0 1.9 **0.20**	2.1 1.9 **0.14**	2.1 2.0 **0.21**	2.1 2.0 **0.23**
ADAS Word Recognition	6.5 6.7 **0.49**	6.5 6.8 **0.82**	6.7 7.1 **0.84**	6.9 7.3 **0.84**	7.3 7.6 **0.67**	7.1 7.5 **0.83**
	3.5 3.3 **0.19**	3.4 3.4 **0.95**	3.4 3.5 **0.28**	3.5 3.3 **0.08**	3.5 3.4 **0.15**	3.6 3.5 **0.33**
MMSE Attention Calculation	3.7 3.2 **0.96**	2.7 2.5 **0.78**	3.2 3.1 **0.38**	2.5 2.3 **0.79**	2.9 2.9 **0.04**	2.3 2.2 **0.49**
	1.7 1.8 **0.44**	1.9 1.8 **0.01**	1.9 1.8 **0.10**	2.0 1.8 **0.02**	1.9 1.8 **0.20**	2.0 1.8 **0.00**
MMSE Language	7.7 7.6 **0.56**	7.7 7.5 **0.94**	7.6 7.5 **0.32**	7.4 7.5 **0.32**	7.3 7.4 **0.14**	7.2 7.3 **0.35**
	1.3 1.4 **0.90**	1.4 1.4 **0.21**	1.4 1.5 **0.68**	1.6 1.5 **0.86**	1.7 1.6 **0.79**	1.8 1.7 **0.90**
MMSE Orientation	6.3 5.7 **0.97**	6.2 5.7 **0.98**	5.7 5.4 **0.60**	5.7 5.4 **0.88**	5.3 4.8 **0.91**	5.3 4.9 **0.96**
	2.4 2.3 **0.38**	2.4 2.3 **0.69**	2.6 2.3 **0.05**	2.7 2.4 **0.07**	2.7 2.4 **0.08**	2.7 2.5 **0.01**
MMSE Recall	0.66 0.68 **0.11**	0.82 0.63 **0.99**	0.80 0.78 **0.16**	0.76 0.74 **0.13**	0.66 0.73 **0.46**	0.67 0.73 **0.56**
	0.96 0.91 **0.89**	1.04 0.91 **0.01**	1.04 0.92 **0.70**	0.99 0.97 **0.87**	1.03 0.91 **0.55**	0.99 0.97 **0.44**
MMSE Registration	2.9 2.9 **0.12**	2.9 2.9 **0.35**	2.9 2.9 **0.73**	2.8 2.8 **0.27**	2.8 2.8 **0.69**	2.8 2.8 **0.47**
	0.5 0.4 **0.88**	0.5 0.4 **0.65**	0.5 0.3 **0.27**	0.6 0.6 **0.73**	0.7 0.5 **0.31**	0.7 0.5 **0.53**
Weight	72 73 **0.17**	71 71 **0.02**	73 72 **0.20**	71 70 **0.63**	73 72 **0.50**	71 71 **0.46**
	16 14 **0.16**	15 14 **0.30**	16 14 **0.23**	16 14 **0.21**	16 14 **0.19**	16 13 **0.09**
Diastolic Blood Pressure	75 76 **0.53**	76 75 **0.67**	76 74 **0.94**	74 75 **0.52**	75 75 **0.00**	75 76 **0.85**
	10 10 **0.33**	10 10 **0.34**	10 10 **1.00**	10 10 **0.50**	10 9 **0.30**	9 10 **0.02**
Systolic Blood Pressure	134 135 **0.78**	135 134 **0.20**	133 133 **0.04**	132 133 **0.47**	133 133 **0.01**	132 133 **0.23**
	17 17 **0.32**	18 17 **0.47**	18 18 **0.08**	17 17 **0.13**	16 17 **0.90**	17 17 **0.71**
Heart Rate	67 67 **0.04**	68 68 **0.02**	67 68 **0.78**	67 68 **0.81**	68 68 **0.69**	67 69 **0.88**
	9 10 **0.39**	10 9 **0.04**	9 9 **0.96**	10 10 **0.93**	9 10 **0.57**	10 9 **0.44**
Alanine Aminotransferase	0.32 0.31 **0.52**	0.31 0.32 **0.16**	0.32 0.31 **0.44**	0.31 0.31 **0.23**	0.31 0.30 **0.55**	0.32 0.32 **0.08**
	0.16 0.12 **0.39**	0.16 0.12 **0.64**	0.21 0.12 **0.33**	0.13 0.12 **0.51**	0.16 0.12 **0.82**	0.16 0.13 **0.92**
Alkaline Phosphatase	1.3 1.3 **0.31**	1.3 1.3 **0.43**	1.3 1.4 **1.00**	1.3 1.4 **1.00**	1.3 1.3 **0.63**	1.3 1.3 **0.23**
	0.4 0.4 **0.98**	0.4 0.4 **0.99**	0.4 0.4 **0.01**	0.4 0.4 **0.10**	0.4 0.4 **0.25**	0.4 0.4 **0.58**
Aspartate Aminotransferase	0.36 0.36 **0.32**	0.36 0.35 **0.56**	0.36 0.36 **0.21**	0.36 0.35 **0.89**	0.35 0.35 **0.11**	0.36 0.35 **0.49**
	0.11 0.09 **0.31**	0.10 0.09 **0.61**	0.11 0.09 **0.63**	0.09 0.09 **0.41**	0.09 0.09 **0.93**	0.09 0.09 **0.80**
Cholesterol	5.5 5.5 **0.33**	5.4 5.4 **0.11**	5.4 5.3 **0.53**	5.4 5.3 **0.90**	5.3 5.2 **0.61**	5.4 5.3 **0.88**
	1.2 1.0 **0.07**	1.1 1.0 **0.33**	1.2 1.0 **0.03**	1.1 1.1 **0.54**	1.2 1.0 **0.02**	1.1 1.0 **0.09**
Creatine Kinase	1.0 1.0 **0.72**	1.0 0.9 **0.99**	0.95 0.95 **0.02**	1.00 0.86 **1.00**	0.93 0.83 **0.95**	0.93 0.92 **0.10**
	0.7 0.6 **0.43**	0.7 0.5 **0.02**	0.65 0.57 **0.83**	0.72 0.50 **0.04**	0.56 0.44 **0.35**	0.58 0.51 **0.86**
Creatinine	0.94 0.95 **0.46**	0.96 0.97 **0.22**	0.97 0.99 **0.63**	0.96 0.97 **0.58**	0.99 0.98 **0.26**	0.99 0.97 **0.50**
	0.23 0.21 **0.97**	0.25 0.22 **0.33**	0.25 0.22 **0.25**	0.25 0.21 **0.34**	0.27 0.22 **0.11**	0.26 0.20 **0.01**
Gamma Glutamyl Transferase	2.4 2.4 **0.17**	2.3 2.3 **0.33**	2.1 2.3 **0.93**	2.3 2.4 **0.22**	2.3 2.4 **0.57**	2.4 2.5 **0.31**
	1.6 1.5 **0.61**	2.4 1.3 **0.83**	1.2 1.4 **0.15**	1.6 1.5 **0.80**	1.7 1.6 **0.88**	2.2 1.9 **0.79**
Hematocrit	0.43 0.42 **0.74**	0.43 0.42 **0.61**	0.43 0.43 **0.30**	0.43 0.42 **0.70**	0.43 0.43 **0.83**	0.42 0.42 **0.31**
	0.04 0.04 **0.17**	0.04 0.04 **0.50**	0.04 0.04 **0.29**	0.04 0.04 **0.56**	0.04 0.04 **0.82**	0.04 0.04 **0.98**
Hemoglobin	14.0 14.0 **0.15**	13.9 13.9 **0.33**	13.9 13.8 **0.32**	13.8 13.8 **0.64**	13.8 13.6 **0.85**	13.8 13.6 **0.92**
	1.2 1.2 **0.74**	1.3 1.2 **0.48**	1.2 1.2 **0.74**	1.3 1.2 **0.70**	1.4 1.3 **0.72**	1.3 1.2 **0.35**
Hemoglobin A1C	5.8 5.8 **0.22**	5.8 5.8 **0.02**	5.8 5.8 **0.17**	5.8 5.8 **0.03**	5.8 5.8 **0.44**	5.8 5.8 **0.45**
	0.8 0.8 **0.94**	0.8 0.7 **0.94**	0.7 0.7 **0.96**	0.7 0.7 **0.85**	0.8 0.7 **0.82**	0.7 0.6 **0.78**
Indirect Bilirubin	0.52 0.52 **0.01**	0.51 0.51 **0.11**	0.53 0.52 **0.30**	0.51 0.49 **0.56**	0.50 0.47 **0.76**	0.53 0.49 **0.89**
	0.25 0.23 **0.52**	0.26 0.24 **0.53**	0.29 0.24 **0.42**	0.27 0.20 **0.28**	0.24 0.21 **0.38**	0.28 0.20 **0.03**
Potassium	4.4 4.3 **0.60**	4.3 4.3 **0.04**	4.3 4.3 **0.30**	4.3 4.4 **0.40**	4.3 4.3 **0.45**	4.3 4.3 **0.15**
	0.4 0.4 **0.35**	0.4 0.4 **0.99**	0.4 0.4 **0.94**	0.4 0.4 **0.26**	0.4 0.4 **0.35**	0.4 0.4 **0.98**
Sodium	1.4 1.4 **0.82**	1.4 1.4 **0.36**	1.4 1.4 **0.82**	1.4 1.4 **0.25**	1.4 1.4 **0.52**	1.4 1.4 **0.08**
	0.0 0.0 **0.61**	0.0 0.0 **0.55**	0.0 0.0 **0.67**	0.0 0.0 **0.60**	0.0 0.0 **0.70**	0.0 0.0 **0.86**
Triglycerides	1.7 1.5 **0.89**	1.5 1.5 **0.05**	1.6 1.5 **0.47**	1.6 1.6 **0.14**	1.5 1.5 **0.05**	1.6 1.4 **0.90**
	1.0 0.8 **0.12**	0.7 0.7 **0.83**	0.8 0.7 **0.76**	0.8 0.7 **0.37**	0.7 0.7 **0.78**	0.8 0.6 **0.09**

FIG. 5. **Agreement between moments for marginal distributions.** For a random CV fold, the mean and standard deviation are compared between the data (the test set for the fold) and synthetic patients produced by the CRBM conditioned on the baseline data, for every time-dependent variable (except dropout) and every time point. The means are compared with a t-test, and the standard deviations are compared with Levene's test. For each, p-values are shown. No significant p-values ($p < 0.05$) are observed after a Bonferroni correction, implying accuracy in the CRBM for these two moments of the marginal distributions. For each variable at each time point, patients with missing data are not included in either the data or CRBM statistical moment calculations. The units are given in Tables I and II.

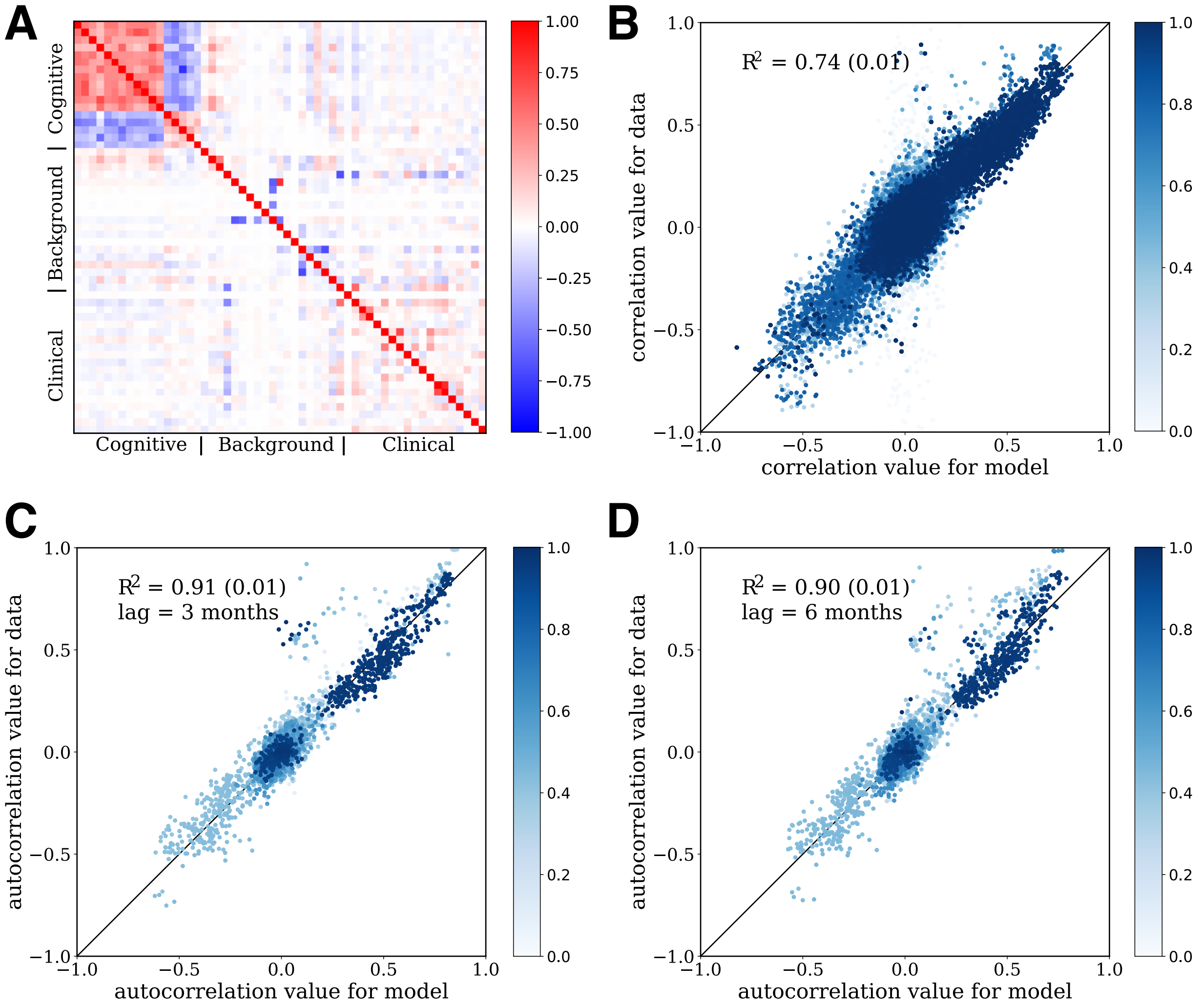

FIG. 6. **Goodness-of-fit**. A) Correlations between variables as predicted by the model (below the diagonal) generating purely synthetic patients and calculated from the data (above the diagonal). Components of the cognitive scores are strongly correlated with each other, but not with other clinical data. B) Scatterplot of observed vs predicted correlations for each time point, over all times. C) Scatterplot of observed vs predicted autocorrelations with time lag of 3 months. D) Scatterplot of observed vs predicted autocorrelations with time lag of 6 months. Color gradient in B-D represents the fraction of observations where the variables used to compute the correlation were present; lighter colors mean more of the data was missing. This figure is a complement to Figure 2 in the main text; in that case the CRBMs were conditioned upon the subject data at baseline, while in this case the models are used purely generatively.

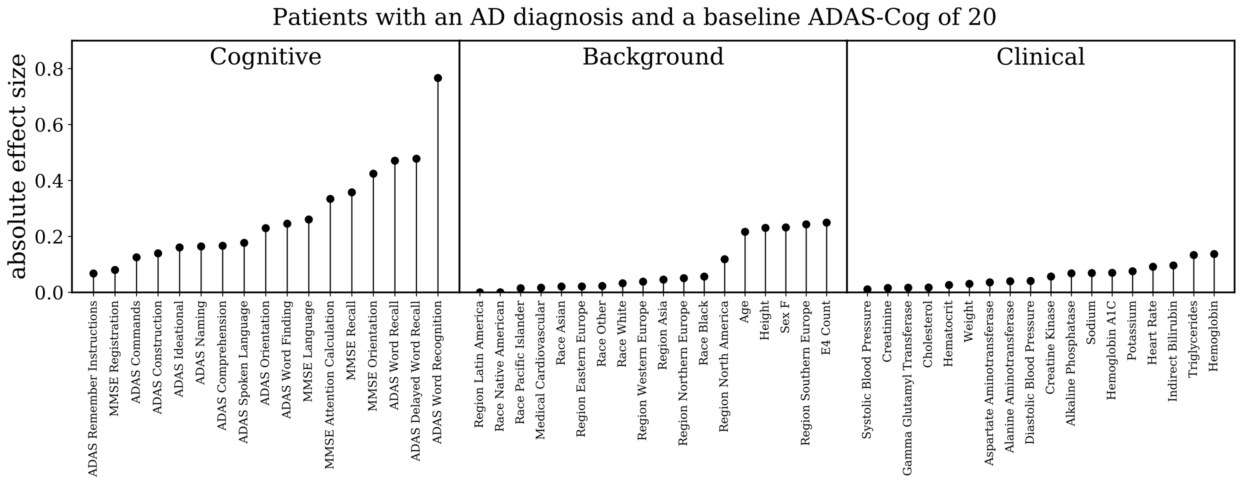

FIG. 7. **Using simulations to interpret prognostic signals for AD progression**. We created a simulated patient population with AD and an initial ADAS score of 20 (typical for AD), and simulated the evolution of each virtual patient for 18 months. The 5% of virtual patients with the largest ADAS score increase were designated "fast progressors" and the bottom 5% of patients with the smallest ADAS score increase were designated "slow progressors". Differences between the fast and slow progressors (the "absolute effect size") were quantified using the absolute value of Cohen's d-statistic. The statistic values are averaged over CV folds. This figure is a complement to Figure 5C in the main text; in that case the effect size for MCI patients with an initial ADAS-Cog of 10 was examined.

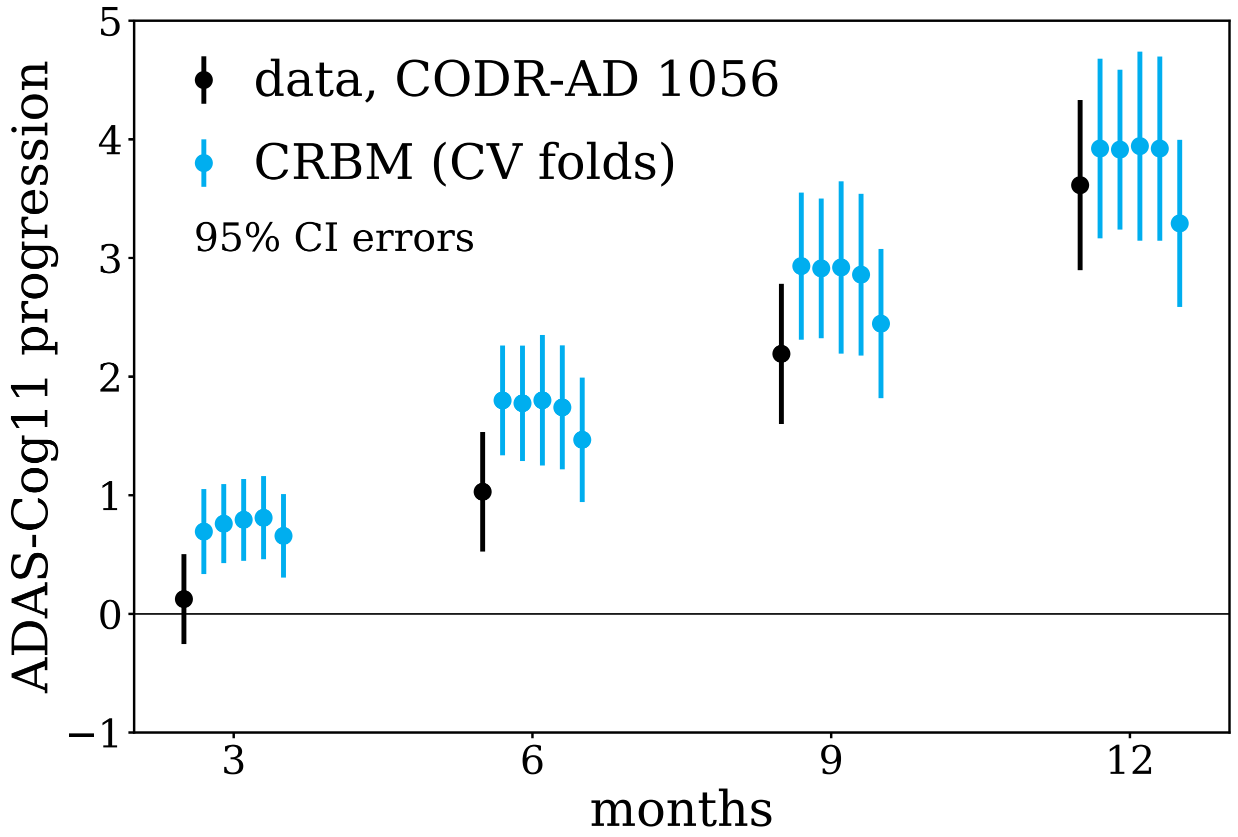

FIG. 8. **The model accurately simulates clinical trials.** The prediction of the model for the progression of ADAS-Cog in a 12-month CODR-AD study is shown. The study is not part of the training data. Predictions for all 5 CV models are shown compared to the progression of the data. The models over-predict progression at the initial 3-month visit, but have good accuracy for later visits. The error bars shown are 95% confidence intervals derived from the standard error of the data and the CRBMs; for each CRBM 100 simulations were used to compute the mean and standard error.

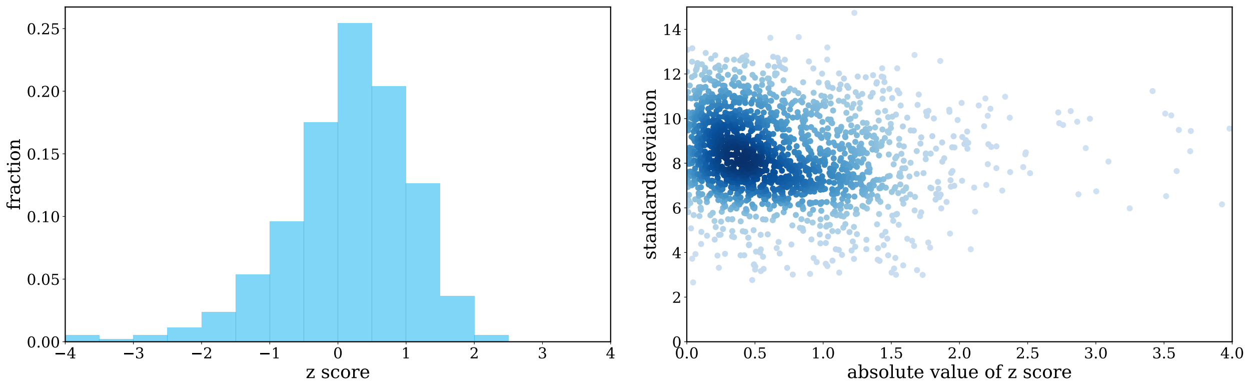

FIG. 9. **Confidence in ADAS-Cog score progression by the CRBM**. For each patient with a valid ADAS-Cog score at baseline and 18 months, the CRBM is used to repeatedly simulate 18-month trajectories. The mean of the ADAS-Cog score changes from these trajectories is the CRBM prediction, and the standard deviation is a measure of the CRBM confidence. These values are used to compute a standard z score for each patient by taking the difference between the CRBM prediction and the true ADAS-Cog score change and dividing by the standard deviation of predictions. These z scores are 0-centered, tend to be fairly normally distributed (A), and do not correlate with the CRBM confidence (B). The color of the points in (B) corresponds to the density; darker points are in a higher-density region. Data from all CV folds aggregated together are shown in these plots.

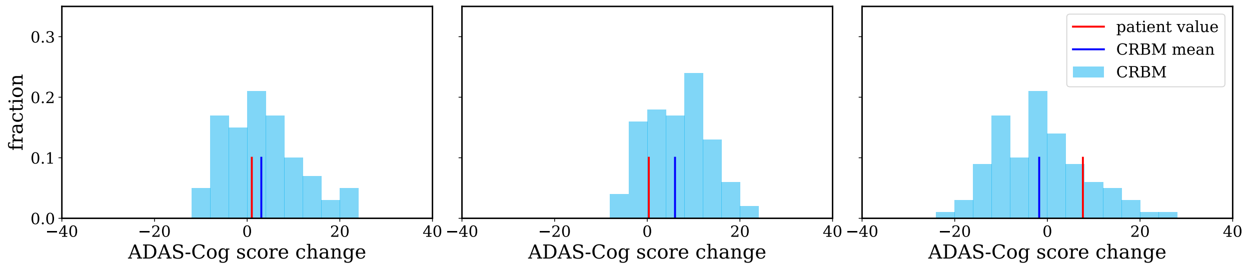

FIG. 10. **Predictions of ADAS score progression for example patients**. Starting with baseline data for 3 example patients, the CRBM was used to predict the change in ADAS score over 18 months. By repeatedly simulating trajectories for each patient, the CRBM provides a set of predictions per patient that forms a probability distribution. The mean of this distribution is the CRBM prediction, which is compared with the true value of the ADAS score change for the patient. The width of the distribution is a measure of the confidence of the CRBM prediction.

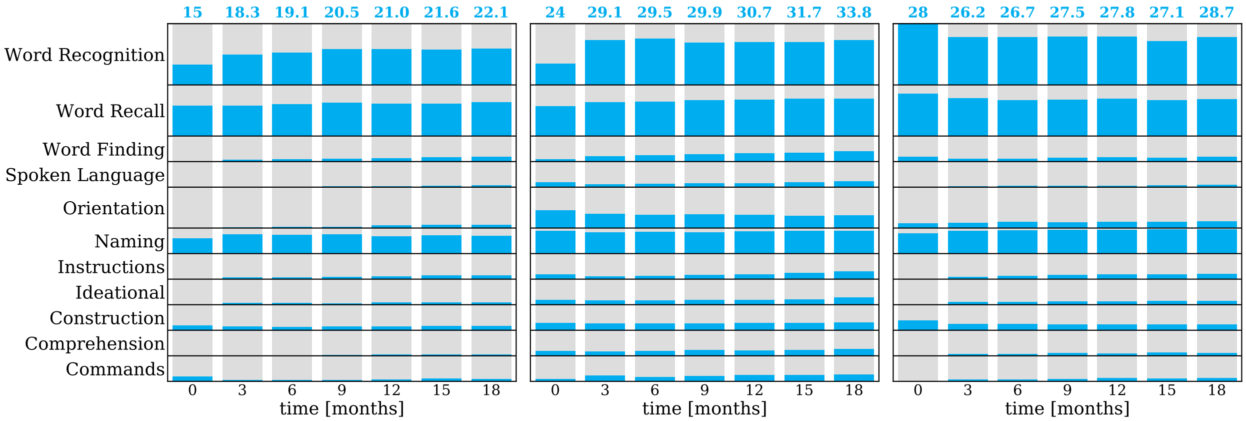

FIG. 11. **Expected evolution of ADAS score components for example patients**. Starting with baseline data for 3 example patients, the CRBM was used to repeatedly simulate 18-month trajectories for each patient. The mean value of each of the ADAS-Cog score components for each time point is shown as a blue bar, demonstrating the ability of the CRBM to simulate the granular ADAS score components. The total mean ADAS score for each time point is shown at the top.

	CRBM	RF(g)	CRBM	RF(g)	CRBM	RF(g)	CRBM	RF(g)	CRBM	RF(g)	CRBM	RF(g)
ADAS Commands	**0.78** (0.02)	0.98 (0.01)	**0.79** (0.02)	0.97 (0.01)	**0.77** (0.01)	0.96 (0.00)	**0.76** (0.03)	0.96 (0.01)	**0.74** (0.04)	0.96 (0.01)	**0.75** (0.01)	0.96 (0.01)
ADAS Comprehension	**0.74** (0.03)	0.97 (0.02)	**0.77** (0.04)	0.99 (0.02)	**0.76** (0.05)	0.97 (0.01)	**0.78** (0.04)	0.98 (0.01)	**0.75** (0.06)	0.96 (0.01)	**0.77** (0.05)	0.97 (0.01)
ADAS Construction	**0.71** (0.03)	0.97 (0.01)	**0.73** (0.03)	0.98 (0.01)	**0.75** (0.03)	0.96 (0.01)	**0.76** (0.02)	0.97 (0.02)	**0.77** (0.02)	0.96 (0.01)	**0.79** (0.01)	0.96 (0.01)
ADAS Delayed Word Recall	**0.52** (0.03)	0.83 (0.04)	**0.52** (0.02)	0.87 (0.03)	**0.55** (0.02)	0.83 (0.04)	**0.54** (0.03)	0.86 (0.01)	**0.59** (0.03)	0.79 (0.03)	**0.61** (0.03)	0.87 (0.04)
ADAS Ideational	**0.78** (0.03)	0.96 (0.02)	**0.77** (0.03)	0.98 (0.02)	**0.75** (0.02)	0.95 (0.01)	**0.75** (0.02)	0.95 (0.01)	**0.73** (0.02)	0.95 (0.01)	**0.75** (0.02)	0.96 (0.01)
ADAS Naming	**0.60** (0.04)	0.95 (0.02)	**0.66** (0.02)	0.96 (0.01)	**0.67** (0.03)	0.94 (0.01)	**0.68** (0.02)	0.95 (0.01)	**0.70** (0.03)	0.94 (0.02)	**0.74** (0.03)	0.96 (0.01)
ADAS Orientation	**0.59** (0.02)	0.89 (0.03)	**0.62** (0.03)	0.90 (0.02)	**0.63** (0.04)	0.87 (0.03)	**0.65** (0.02)	0.89 (0.01)	**0.65** (0.04)	0.86 (0.02)	**0.68** (0.02)	0.90 (0.03)
ADAS Remember Instructions	**0.74** (0.03)	0.97 (0.02)	**0.74** (0.02)	0.97 (0.01)	**0.75** (0.03)	0.95 (0.01)	**0.78** (0.02)	0.96 (0.01)	**0.78** (0.02)	0.95 (0.01)	**0.78** (0.02)	0.96 (0.01)
ADAS Spoken Language	**0.70** (0.06)	0.98 (0.01)	**0.73** (0.05)	0.99 (0.02)	**0.75** (0.06)	0.98 (0.00)	**0.76** (0.02)	0.98 (0.01)	**0.77** (0.04)	0.98 (0.02)	**0.78** (0.03)	0.98 (0.01)
ADAS Word Finding	**0.69** (0.02)	0.96 (0.00)	**0.73** (0.03)	0.98 (0.02)	**0.76** (0.03)	0.97 (0.01)	**0.75** (0.02)	0.97 (0.01)	**0.75** (0.02)	0.96 (0.02)	**0.78** (0.01)	0.97 (0.01)
ADAS Word Recall	**0.54** (0.03)	0.85 (0.03)	**0.54** (0.03)	0.88 (0.03)	**0.59** (0.01)	0.85 (0.04)	**0.59** (0.03)	0.87 (0.00)	**0.61** (0.01)	0.82 (0.03)	**0.61** (0.02)	0.88 (0.04)
ADAS Word Recognition	**0.72** (0.02)	0.89 (0.02)	**0.78** (0.01)	0.93 (0.01)	**0.76** (0.02)	0.89 (0.03)	**0.77** (0.02)	0.92 (0.01)	**0.76** (0.01)	0.89 (0.02)	**0.74** (0.02)	0.91 (0.03)
MMSE Attention Calculation	**0.77** (0.04)	0.98 (0.02)	**0.78** (0.01)	0.96 (0.02)	**0.84** (0.04)	0.99 (0.01)	**0.83** (0.01)	0.97 (0.01)	**0.84** (0.04)	0.98 (0.01)	**0.85** (0.02)	0.97 (0.01)
MMSE Language	**0.80** (0.02)	0.99 (0.03)	**0.84** (0.03)	1.01 (0.01)	**0.77** (0.04)	0.99 (0.01)	**0.77** (0.04)	0.97 (0.01)	**0.75** (0.04)	0.99 (0.01)	**0.79** (0.02)	0.98 (0.01)
MMSE Orientation	**0.68** (0.05)	0.95 (0.02)	**0.72** (0.04)	0.94 (0.01)	**0.76** (0.04)	0.96 (0.03)	**0.77** (0.03)	0.95 (0.01)	**0.80** (0.03)	0.97 (0.01)	**0.80** (0.03)	0.96 (0.02)
MMSE Recall	**0.85** (0.02)	0.99 (0.02)	**0.87** (0.02)	0.98 (0.01)	**0.85** (0.03)	0.98 (0.02)	**0.88** (0.03)	0.98 (0.01)	**0.87** (0.05)	0.98 (0.01)	**0.89** (0.01)	0.98 (0.01)
MMSE Registration	**0.98** (0.04)	1.00 (0.01)	**0.99** (0.05)	1.02 (0.00)	**0.97** (0.10)	1.02 (0.04)	**0.97** (0.02)	1.01 (0.00)	**0.97** (0.02)	1.01 (0.02)	**0.97** (0.02)	1.02 (0.02)
Weight	**0.24** (0.01)	0.55 (0.04)	0.39 (0.02)	**0.28** (0.01)	**0.47** (0.02)	0.56 (0.05)	0.55 (0.02)	**0.32** (0.01)	0.60 (0.04)	**0.55** (0.05)	0.64 (0.04)	**0.36** (0.01)
Diastolic Blood Pressure	**0.83** (0.01)	0.86 (0.02)	**0.88** (0.01)	0.92 (0.02)	**0.91** (0.01)	0.92 (0.02)	**0.93** (0.01)	0.95 (0.02)	0.94 (0.01)	**0.92** (0.01)	0.96 (0.01)	**0.95** (0.02)
Systolic Blood Pressure	**0.80** (0.01)	0.80 (0.02)	0.87 (0.01)	**0.86** (0.03)	0.92 (0.02)	**0.88** (0.03)	0.94 (0.01)	**0.87** (0.02)	0.95 (0.01)	**0.88** (0.02)	0.97 (0.01)	**0.90** (0.03)
Heart Rate	0.92 (0.09)	**0.82** (0.02)	0.95 (0.06)	**0.90** (0.02)	0.97 (0.03)	**0.92** (0.03)	0.98 (0.02)	**0.93** (0.02)	0.99 (0.01)	**0.93** (0.01)	0.98 (0.01)	**0.93** (0.02)
Alanine Aminotransferase	**0.75** (0.02)	0.97 (0.01)	**0.81** (0.07)	0.98 (0.01)	**0.86** (0.02)	0.98 (0.01)	**0.85** (0.03)	0.97 (0.01)	**0.91** (0.03)	1.02 (0.02)	**0.90** (0.04)	0.99 (0.03)
Alkaline Phosphatase	**0.77** (0.23)	0.99 (0.02)	**0.82** (0.19)	1.06 (0.05)	**0.88** (0.13)	1.02 (0.01)	**0.92** (0.08)	0.99 (0.01)	**0.92** (0.10)	0.99 (0.02)	**0.93** (0.07)	1.06 (0.09)
Aspartate Aminotransferase	**0.77** (0.03)	0.99 (0.01)	**0.82** (0.05)	0.99 (0.02)	**0.86** (0.01)	0.99 (0.01)	**0.88** (0.02)	0.99 (0.01)	**0.92** (0.03)	1.04 (0.03)	**0.93** (0.03)	1.00 (0.03)
Cholesterol	**0.59** (0.04)	0.97 (0.01)	**0.67** (0.04)	0.98 (0.01)	**0.75** (0.04)	0.98 (0.01)	**0.78** (0.03)	0.97 (0.01)	**0.84** (0.02)	0.98 (0.01)	**0.89** (0.02)	0.99 (0.01)
Creatine Kinase	**0.93** (0.03)	1.00 (0.01)	**0.93** (0.01)	0.99 (0.01)	**0.95** (0.01)	0.99 (0.00)	**0.96** (0.01)	0.99 (0.02)	**0.97** (0.02)	1.01 (0.02)	**0.97** (0.02)	0.99 (0.01)
Creatinine	**0.49** (0.03)	0.96 (0.02)	**0.60** (0.04)	0.97 (0.02)	**0.65** (0.03)	0.98 (0.02)	**0.68** (0.03)	0.95 (0.02)	**0.74** (0.02)	0.98 (0.01)	**0.76** (0.03)	0.97 (0.01)
Gamma Glutamyl Transferase	**0.50** (0.05)	0.98 (0.02)	**0.74** (0.16)	1.04 (0.04)	**0.66** (0.06)	1.01 (0.03)	**0.75** (0.05)	0.99 (0.02)	**0.82** (0.09)	1.03 (0.03)	**0.83** (0.06)	1.05 (0.10)
Hematocrit	**0.60** (0.03)	0.93 (0.03)	**0.64** (0.02)	0.95 (0.01)	**0.66** (0.03)	0.96 (0.02)	**0.70** (0.03)	0.94 (0.01)	**0.71** (0.02)	0.95 (0.02)	**0.77** (0.01)	0.95 (0.02)
Hemoglobin	**0.51** (0.02)	0.93 (0.02)	**0.59** (0.01)	0.94 (0.03)	**0.63** (0.05)	0.95 (0.02)	**0.67** (0.01)	0.93 (0.01)	**0.69** (0.02)	0.94 (0.01)	**0.73** (0.01)	0.94 (0.02)
Hemoglobin A1C	**0.40** (0.07)	0.99 (0.01)	**0.53** (0.11)	0.99 (0.04)	**0.60** (0.12)	0.99 (0.01)	**0.62** (0.08)	0.99 (0.01)	**0.67** (0.08)	0.96 (0.01)	**0.66** (0.08)	0.99 (0.01)
Indirect Bilirubin	**0.72** (0.06)	0.98 (0.01)	**0.83** (0.05)	1.00 (0.00)	**0.86** (0.06)	1.00 (0.01)	**0.90** (0.04)	0.98 (0.01)	**0.90** (0.03)	1.00 (0.02)	**0.92** (0.02)	0.99 (0.01)
Potassium	**0.97** (0.01)	0.99 (0.02)	**0.98** (0.01)	1.00 (0.01)	**0.98** (0.01)	1.00 (0.01)	**0.98** (0.01)	1.00 (0.01)	1.00 (0.01)	**0.99** (0.01)	**0.99** (0.01)	1.01 (0.01)
Sodium	**0.96** (0.01)	0.99 (0.00)	**0.96** (0.01)	0.99 (0.01)	**0.97** (0.02)	1.00 (0.00)	**0.96** (0.01)	1.00 (0.01)	**0.97** (0.01)	1.00 (0.01)	**0.98** (0.01)	0.99 (0.01)
Triglycerides	**0.83** (0.05)	0.99 (0.02)	**0.91** (0.02)	0.99 (0.01)	**0.92** (0.02)	0.99 (0.02)	**0.93** (0.02)	0.99 (0.01)	**0.95** (0.02)	1.00 (0.02)	**0.96** (0.01)	0.99 (0.03)
	3 months		6 months		9 months		12 months		15 months		18 months	

FIG. 12. **The model accurately forecasts across variables.** Relative errors of the model (CRBM) and a "global" random forest (RF(g)) trained to predict the value of all variables at a single time point. The root mean square (RMS) errors are scaled by the standard deviation of the data to be predicted. Predictions are shown for every time-dependent variable except dropout. At each time point and for each variable, the better of the random forest and CRBM predictions is shown in bold, with uncertainties across CV folds shown in parentheses below the mean value. The CRBM strongly outperforms the global random forest. This figure is a complement to Figure 3, where here the random forest is trained to predict all variables at a time point instead of having separate random forests for each variable.
